# Abstracts of papers presented at the International Symposium on Laboratory Automation and Robotics (October 1993)

**DOI:** 10.1155/S1463924694000015

**Published:** 1994


Journal of Automatic Chemistry, Vol. 16, No. 1 (January–February 1994), pp. 1–24

### Abstracts of papers presented at the International Symposium on Laboratory Automation and Robotics (October 1993)

*The following are the abstracts of papers and posters presented at ISLAR 1993 (the 11th meeting in the series), which was held in Boston from 17–20 October 1993. Once again, the Editor wishes to thank the organizer of the ISLAR meetings, Zymark Corporation, for permission to reproduce the abstracts and bring them to the attention of a wider audience.*

*The next ISLAR will be held from 16–19 October 1994 in Boston. Details are available from: Christine O'Neil, Zymark Corporation, Zymark Center, Hopkinton, MA 01748, USA. Tel.: 508 435 9500, ext. 2224; fax 508 435 3439.*

### Laboratory automation

### Keynote 1: Laboratory automation: a challenge for the 1990s

*Claude Mordini, Rhone Poulenc SA, Paris, France*

At the beginning of the 1980s, laboratory automation was mostly dedicated to robotics, which was mainly driven by and developed in pharmaceutical/biological applications and then extended to other areas such as chemical, quality control, and environmental analysis. Robotic systems emerged which integrated unit operations, transfers and measurements.

The concept of automation considerably matured in the second half of the 1980s—first, because of the need to manage more and more data, and second because of the development of new computer science technologies. This keynote presentation discussed the impact of information systems, data bases, knowledge bases, LIMS, artificial intelligence, experimental design and modelling on laboratory automation. Through laboratory automation, we can achieve more integration of laboratory functions. The whole should be accessed and managed through a single station and interface.

### Keynote 2: Bioanalytical automation: history and future plans

*Raymond H. Farmen, Bristol-Myers Squibb, Princeton, NJ*

Bioanalysis involves quantifying the concentration of drugs and their metabolites in biological fluids and is the cornerstone of pharmacokinetics and pharmacodynamics. Within the past two decades, bioanalysis has been transformed from a tedious manual operation to an automated and computer intensive discipline. There are at least nine distinct steps involved in bioanalysis. Unfortunately, only four of these steps have been automated, and the seams between these automated steps are often poorly managed. As the importance of pharmacokinetics in the drug registration process has increased, the number of samples requiring bioanalysis has increased exponentially. A plan for a fully automated bioanalytical laboratory was presented which has the capacity to process 400 000 samples/year.

### Laboratory workstations

### A semi-automated quantitative analytical method for the determination of anti-hypertensive drug candidates, CGP 48933 and/or CGP 48369 in human plasma using high performance liquid chromatography

*Linda A. Brunner, Development Department, Pharmaceuticals Division, Ciba-Geigy Corporation, Ardsley, NY*

A semi-automated method utilizing a BenchMate solid phase extraction (SPE) laboratory workstation has been developed and validated for simultaneously quantifying concentrations of two new anti-hypertensive drug candidates (CGP 48933 and/or CGP 48369) in human plasma. Following the precipitation of plasma proteins, the workstation adds the internal standard (CGP 48791), adjusts the pH (acidic) through the addition of buffer, and elutes the compounds of interest from 3-ml cyclohexyl (CH) SPE cartridges with methanol. The eluents are concentrated on a TurboVap® evaporation station for subsequent reversed-phase, high performance liquid chromatographic (HPLC) analysis. Separation is achieved on a 5-mm, Inertsil ODS-2 (4·6 × 150 mm) column at 40°C with fluorescence detection of the drugs and internal standard at $l_{EM}$ = 265 nm and $l_{EM}$ = 378 nm. Recovery and reproducibility assessments indicate good accuracy (overall mean relative recovery of 94·9%) and precision (coefficient of variation, CV ≤ 12·8%) over the CGP 48933 concentration range of 50 to 5000 ng/ml, with a quantification limit of 50 ng/ml. Similar values were determined for CGP 48369, with an accuracy of 91·9% and precision ≤15·5%, over the same concentration range. The method has been successfully applied to a pharmacokinetic study in which normal volunteers received a single, oral dose of 160-mg CGP 48933. Up to 100 samples can be analysed during one run of the workstation, and up to 50 samples can be processed on the TurboVap evaporator at one time.

© 1994 Zymark Corporation

### Evaluation of solid phase extraction of antiviral nucleoside analogues from human plasma utilizing BenchMate robotic workstation

*Patrick J. Faustino, US Food and Drug Administration, Laurel, MD*

The existence of pathological agents in human plasma has provided added incentive to search for ways to reduce or eliminate manual intervention in the sample preparation process. Utilizing automation may provide the means for added safety, efficiency and quality of results.

Determination of plasma levels of antiviral nucleoside analogues such as AZT (axidothymidine), DDC (dideoxycytidine) and DDI (dideoxyinosine) in an efficient and safe manner, is crucial to both the research and analytical laboratory. Methods development of solid phase extraction of antiviral nucleoside analogues utilized a BenchMate Workstation for extraction and a Hewlett Packard 1090M HPLC for on-line separation and detection. Manual and automated extraction to each nucleoside analogue were evaluated to determine the efficiency and precision of each technique.

### Automating a manual solid phase extraction method

*Lynn Jordan, Zymark Corporation, Zymark Center, Hopkinton, MA 01748; and Gerald Long, United Chemical Technology, Inc., Horsham, PA 19044*

Solid Phase Extraction (SPE) is often the method of choice for sample preparation because of the high degree of specificity available to the user in developing methodologies. Variations in vacuum box performance can cause variable recoveries with a manual SPE method. Automating a manual SPE method can help reduce the variability of recoveries by using precise, independently controlled flow rates for the condition, load, rinse and elution steps.

This paper illustrated the process of transferring a manual SPE method to an automated workstation. Automation of an SPE method involves a few steps to ensure that the automated method will be rugged. The first step is to rule out the presence of any interferences in the results of the automated SPE method. Second, the automated method is optimized to obtain the highest recovery possible. Third, the method should be checked for carry-over by running a standard followed by a blank. Finally, an automated method can be optimized for throughput as long as none of the positive attributes of the automated method is lost.

### Microplates A

### Development of an automated high-throughput assay system for the discovery of compounds active against the AIDS virus

*Rudi Pauwels, Jan Desmyter and E. De Clercq, Rega Institute for Medical Research, Leuven, Belgium*

It is currently estimated that approximately 10 M people are infected with human immunodeficiency virus (HIV), the causative agent of AIDS. Managing and counteracting the HIV/AIDS pandemic therefore requires effective antiviral therapy. It is generally accepted that if one could block HIV replication *in vivo*, further impairment of the immune status in HIV-infected patients would slow down and possibly stop.

To this end the authors started a project to find inhibitors of HIV replication *in vitro* in 1985. A primary screening assay that filters out the 'interesting compounds' was developed, i.e. compounds which inhibit HIV replication at concentrations which do not impair the normal cellular functions of the host. The protective effects against HIV, as well as the host cell cytotoxicity of the compounds were quantified with the tetrazolium dye MTT. This dye is taken up and metabolized only in living cells. It thereby generates a colour which can be measured spectrophotometrically.

As the number of collaborations increased, the results and the information of the growing number of experiments had to be rapidly generated and shared with the other project members located in different parts of the world. Therefore, the primary screening assay was automated using microtitre equipment and laboratory robotics (Biomek 1000 Robot-workstation and Zymate II robot). The laboratory automation was complemented by an information management system developed in-house for data capture, processing, reporting and storage. The software also contains various data validation, quality control and joblist generation components. Currently about 300 microtitre plates are processed per week.

The automation played an important role in the discovery of several potent and highly selective HIV inhibitors, some of which are now investigated in HIV-infected patients. It also limited the type and duration of exposure of the laboratory staff to potentially biohazardous materials.

### Progress in automating mapping of the human genome

*M. M. Blanchard, F. W. Burough, D. D. Sloan and V. Nowotny, Washington University School of Medicine, Center for Genetics in Medicine, St. Louis, MO*

The Human Genome Project is an international effort to establish a map of ordered landmarks, Sequence Tagged Sites (STS), along the human genome that consists of about 3 billion basepairs. Through biochemical advances such as the Polymerase Chain Reaction (PCR), and the use of Yeast Artificial Chromosomes (YAC), this project has become feasible. Coverage of the genome with about one STS every 100 kilobase is in progress. The Yeast Artificial Chromosome allows for maintenance and propagation of the human DNA since each YAC contains a piece of human DNA up to lengths exceeding a million base pairs. PCR amplifies short tracts of specific DNA from within a very complex mixture of DNA sequences. To obtain sufficient chromosomal maps of the genome (one STS per 100 kb), a major portion of the Human Genome Project will involve repeated PCR screening of YAC libraries for specific sequences, STSs (about 30 000), and the use of this information to provide an order to the YAC clones.

To meet this great demand and to reduce the labour involved, the Technical Development Laboratory of the Center for Genetics in Medicine has developed a robot-aided screening strategy. The hardware consists of a robotic workstation which includes the use of a BIOMEK 1000 system (Beckman Instruments, Palo Alto, CA) for automated setup of PCR reactions, the design and building of a thermocycler capable of handling 576 samples in over 3 hours, and a storage unit for easy access to the DNA of over 60 000 yeast clones. In addition, the development of uniform PCR conditions for all STSs, and the use of a sophisticated combinatorial DNA pooling scheme, has made recursive screening of YAC libraries much quicker and less labour intensive. Indeed, after several months of use, STS running throughput has increased in speed by a factor of four to five fold per technician. Currently, our major effort involves the assembly of software for the control of this machine to allow a completely automatic screen integrating thousands of pipetting steps and the transfer and running of thousands of PCR reactions each day.

### A flexible robotic workstation for microplate assays

*William S. Fillers and Diana K. Cohen, Sandoz Research Institute, Sandoz Pharmaceuticals Corporation, E. Hanover, NJ*

Dedicated robotic systems designed for large-scale screening approaches to drug discovery often have limited ability to rapidly reconfigure for alternate applications. The authors described successes and challenges in the development of a general purpose, robotized workstation for microplate-based assays. The practical aspects of time and cost effectiveness for robotic assay development were discussed in relation to overall considerations of laboratory response time and resource intensity. Specific examples were drawn from microplate procedures to highlight some problem-solving required for the creation and successful operation of a workstation capable of addressing the need for running a wide variety of microplate screens on a single platform.

### Validation

### Complete validation of the Zymark Tablet Processing Workstation—where no man has gone before

*Stephen Scypinski, Theodore Sadlowski and Linda B. Clark, Pharmaceutical Analysis Research and Development, Hoffmann-La Roche Inc., Nutley, NJ*

The increasing utilization of robotics in the pharmaceutical analysis laboratory is dependent on the ability of such automated systems to meet the rigorous criteria set forth by GMP compliance. Such criteria include the completion of an extensive set of validation trials. To most analysts involved with the use of robotics, this is not surprising. However, the Food and Drug Administration (FDA) has recently extended their expectations of validation to computer systems and computer-controlled equipment. Because a robotic system is a 'hybrid' composed of a computer as well as conventional analytical hardware, it must be validated in accordance with protocols for both areas. As computer validation is a relatively new field, very few precedents have been set. It is therefore the responsibility of the individual company to devise a validation plan for computerized systems. At Hoffmann-La Roche, this plan includes the validation for systems such as the Zymark Tablet Processing Workstation (TPW).

Recently, the authors have focused the activities of their robotics laboratory on developing and performing a complete and thorough validation of the TPW. The validation plan or outline is composed of several elements. These are:

(1) Physical performance testing of each component of the TPW.
(2) Validation of the computer software according to a corporate protocol.
(3) Validation of the final method, encompassing both the hardware and software.

The presentation discussed the validation strategies that have been implemented in certifying the performance of the TPW.

### Concepts and strategies in the validation of robotics methods

*Edward A. Mularz, Physical and Analytical Chemistry Research & Development, Schering Plough Research Institute, Kenilworth, NJ*

This presentation discussed the author's approach to robotic validation and covered several key areas necessary to implement a validated robotic system: developing the manual method; automating the manual method; documentation of both in-house developed programs and Zymark supplied programs; single sample and full system testing; back-up and archiving; boundary and error testing; software version change control; system description and arrangement; calibration of modules; system implementation authorization; and personnel training records. Several robotic procedures have been successfully implemented and validated for drug substance in rodent feed, solid and semi solid dosage forms. These automated methods were documented and validated using standard operating procedures for automated and computer systems.

### Continuous validation of a robotic method for analysis of conjugated estrogens by capillary GC

*Mary E. LaBrecque and Ronald G. Anstey, Analytical Research and Services, Wyeth Ayerst Laboratories, Rouses Point, NY*

Once a robotic system is validated, it must continue to maintain the same precision, accuracy, and reproducibility under continuous daily operation. The two master lab stations on the Zymate XP robot perform six dilutions. Of these six dilutions, three are 'volumetric' and are critical to the authors' application. It was decided to continuously monitor these additions gravimetrically. Feedback loops were written to check volumes after each addition. The volumes are also checked before the sample is injected. Any sample not completed is noted on the post-run report.

This presentation outlined the reasons for continuous validation, how it was implemented, and the results that have been obtained.

### Quality assurance in automated procedures

*Alexander J. Koller, Koller Computer Technologies, Inc., Woodbury, CT*

The need for validation and verification of automated procedures is well known in the laboratory automation arena. What has been difficult to attain is how to improve the quality of automated procedures and how to prove their correctness are implemented. The scope of this problem extends from a small sample preparation robotic work cell to large scale automated systems.

This paper detailed the necessary steps for improving quality assurance during the development cycle by concentrating on designing, developing and testing for quality. Quality does not happen by accident. It is a result of extensive planning, standardization, and then the implementation of the plan and the accepted standards. A discussion of quality assurance would not be complete without discussing system entomology, its associated costs and its impact on validation efforts.

Validation can be a complicated process. If validation efforts are successful then they prove the accuracy, reliability, and general high quality of your system. However, this isn't always the case. Since there are multiple methods of validation, this paper reviewed these methods as they apply to the key issues:

(1) Proof of correctness.
(2) Documentation.

Validation methods will be different depending on whether or not the automated procedure is designed and implemented in-house or is purchased as a 'turn-key' solution. The validation of computer hardware and computer software was discussed with case study examples.

### Automated implementation strategies

### Automation in motion: moving and rebuilding robotics systems

*Bruce Kropscott, James Ormand, Shoreh Shabrang, Timothy Meill and Cynthia Peck, The Dow Chemical Company, Health and Environmental Sciences (H & ES), Midland, MI*

Historically, the three robotics systems in H & ES Analytical Chemistry operated in separate laboratories. Recent building renovation provided an opportunity for these systems to be modernized and consolidated into one laboratory. The challenge was twofold: (1) disassemble and rebuild the systems into functional units; and (2) design a new laboratory with robotics in mind—both modular and mobile.

Originally, the three robotics systems (one Zymate® II-Py Technology and two custom Zymate IIs) had different programmers, some redundant functional attributes, and some specialized hardware. All were in different stages of development. A team approach was used to focus on the similarity of applications and designed two systems with identical capabilities. These changes enabled the use of the robotic equipment to be maximized and for the third system to be redeployed for dedicated use in another group.

Several challenges were encountered in moving when 5′ × 7′ system tables through 3′ doorways into a laboratory still under construction. Electrical power supplies, air and cylinder-gas supplies, computer network hookups, waste disposal facilities, and accessory equipment location were designed with robotics specifically in mind. The advantages of making older systems compatible with newer technology outweighed the constraints of system reconstruction.

This paper described: (1) the challenges of relocating robotics systems; (2) considerations in designing a robotics laboratory; (3) benefits and constraints of upgrading robotics system; and (4) the philosophy of the authors' design in conjunction with their vision for automation.

### Emerging automation strategies

*Edward G. Kanczewski, Parke-Davis Pharmaceutical Research Division, Warner-Lambert Company, Morris Plains, NJ 07950*

Today's submissions to government regulatory agencies require more in-depth investigations and documentation. As a result, laboratories are being asked to conform to more detailed and stringent guidelines. The need for reliable, high-quality data, quick sample turn around time, improved sample scheduling/control and enhanced documentation are goals for which every laboratory strives.

Facilities and techniques such as the integrated laboratory, automtion, robotics and artificial intelligence are in the process of evolution. Incorporation of these concepts into the laboratory will help an organization operate more efficiently and remain competitive. This presentation encompasses an overview of the status of these automation strategies.

### The application architect

*James R. Ormand and Cynthia N. Peck, The Dow Chemical Company, Health and Environmental Sciences, Midland, MI*

Laboratory robots are often dedicated to processing high volumes of one type of sample with high throughput. Analysts face the challenge of implementing robots to process diverse sample sets, while maintaining high throughput. To design an application for each sample type is time consuming, uses a large amount of computer memory, and requires a robotics programmer. However, by taking advantage of the laboratory unit operations that are common to processing most of the authors' sample types, a menu-driven architect program was developed that allows the user to build a sample specific application. This architect program prompts the user for all of the variables necessary to build an application, such as the parameters for vortex extraction and mixing, liquid transfer and dilution, and LC analysis parameters. The sample specific application can then be stored as a parameter file instead of redundant program code. This program has benefited the authors' laboratory by:

(1) Reducing application development time.
(2) Allowing multiple users to benefit from robotics.
(3) Providing standard detailed documentaion for GLP requirements.
(4) Conserving dictionary space.

In this example, an application architect program is used with a Zymate® robot to build feed extraction applications, however, this concept can be used to capitalize on the commonality of many laboratory processes. This paper discussed the strategies for the design, details of this application, and the possibilities for future development.

### Managing laboratory automation

### Decentralized management of laboratory automation: a contrarian approach

*Don Chambers, Schering-Plough Research Institute, Kenilworth, NJ*

It is generally believed, as noted during the Managing Laboratory Automation session of ISLAR 91, that successful robot users have dedicated centralized robotic groups. While such a generalization holds some merit historically, the availability of newer, more user-friendly robots and workstations in recent years and a more computer literate work force today is changing the way automation may be managed.

Decentralization recognizes robots and workstations as additional tools for all analysts, not a select few. Such an approach initiates involvement and education of more staff with respect to automation. This approach further ushers the development of automated methods instead of the automation of manually developed methods. Decentralization also provides local control of resources to address the priorities of a specific functional group within the department.

Both a vision of the future, as well as a look at the past, should be considered when determining how to manage robotic and other means of automation. This presentation discussed decentralization management of robots as currently applied and envisioned in a large analytical R & D department.

### The role of the automation development group in analytical research and development at Du Pont Merck

*John C. Lynch, Jonathan S. Green, Paul K. Hovsepian, Kathleen L. Reilly and Joseph A. Short, The Du Pont Merck Pharmaceutical Company, Wilmington, DE*

Laboratory robotics has been firmly established in many non-QC labs as a valuable tool for automaticing pharmaceutical dosage form analysis. Often a single project or product line is used to justify an initial robot purchase, thus introducing robotics to the lab for the first time. However, to gain widespread acceptance within the lab and to justify further investment in robotics, existing robots must be used to develop analyses for existing manual methods as well as new projects beyond the scope of the original purchase justification. The Automation Development Group in Analytical Research and Development is a team of analysts primarily devoted to developing new methods and adapting existing methods for the robot. The team approach developed the expertise and synergy necessary to significantly expand the contribution of robotics to automation in the authors' laboratory.

### Key issues for establishing a robotics laboratory in the pharmaceutical industry

*Steve Conder, Bristol-Myers Squibb, N. Brunswick, NJ*

The Analytical Research and Development Department of Bristol-Myers Squibb has a laboratory dedicated to robotic analysis of solid dose forms. It consists of eight individuals responsible for nine robotic systems. The laboratory is dedicated to the support of Phase III stability studies that require dissolution, potency, content uniformity and Karl Fischer moisture assays. The group performs about 15–20 000 assays per year for approximately six long-term stability programmes. The key issues for success were personnel selection, methods development (method transfer), routine assays, documentation, validation, training and support services. The laboratory's experiences over the last four years have exposed both strengths and weaknesses in these areas. Future success depends upon improving our flexibility and response time to clients. This presentation discussed the key issues that helped establish the laboratory and the future issues important to continued success.

### Prejudice, segregation and immigration laws—Integration of the robot into laboratory society

*Norman E. Fraley, Jr., Express Analytic, Downers Grove, IL*

This presentation addressed some serious issues about personnel morale, fears and hopes associated with and attributed to the new lab tech robot. The introduction of the laboratory robot into the laboratory from a managerial perspective was discussed. Human-rights and robot-rights issues were identified and addressed. Real world examples of how the integration of two high throughput robots affected the routine of a major industrial food laboratory were discussed.

### Microplates B

### Validation of a robotic system for microplate ELISA

*Mary Jordan Monahan and Theresa J. Giampaglia, Lederle Praxis Biologicals, Pearl River, NY*

Validation of the author's system has been achieved by:

(1) Calibration of the various modules that add, aspirate or dispense measured amounts of fluids between tubes and microplates, for example RAM.1 adds 100 µl to each well of the microplate.
(2) Verification of positional directions outlined in the software, for example SINGLE.TIP.SYRINGE places sample #95 in position G10 on each microplate.
(3) Confirmation of assay ruggedness, shown when robotic assay results compare to those of the manually performed assay with acceptable accuracy and precision.

The full validation was performed, documented and accepted once the robot was assembled and operational, again after the robot was relocated and then again after the application had been changed.

The fluid handling portion of the validation is performed before each run. One 15 min routine verifies the following:

(*a*) RAM.1 adds 100 µl to each well of the microplate.
(*b*) The Eight Channel Hand removes 100 µl from the appropriate sample, adds 100 µl to the correct row of the microplate and dilutes the sample on the microplate.
(*c*) The Single Tip Syringe removes 100 µl from the correct sample tube, adds it to the corresponding well on the microplate and dilutes on the microplate.

The above microplate is read on a microplate reader and the results compared to the parameters set during the original validation. If the results meet or exceed the requirements of the test the operator may proceed with the assay as scheduled. If the results do not meet the requirements of the test; the reason for the failure must be determined, corrected, and a passing plate produced before the assay can be performed.

### High throughput screening for novel anti-inflammatory drug leads

*Michelle Palmer, Genetics Institute, Cambridge, MA*

Genetics Institute is screening for anti-inflammatory drug lead candidates, utilizing Zymark robotic microplate technology. The approach demands high throughput for multiple targets, therefore requiring numerous automated systems with a very high assay capacity. This presentation described the approach and technology utilized in achieving this goal.

### Hands-free polymerase chain reaction

*Larry D. Sutton, Werner W. Wilke and Mary Kay Pappas, University of Iowa, Department of Pathology, Iowa City, IA*

Few papers have been published to date concerning automation of the now legendary polymerase chain reaction (PCR). Of those that have been published, all require some manual intervention during the experiment and none have evaluated performance, especially with regard to rates of contamination. In this paper, the authors reported the successful development of a robotically-automated PCR system.

A Zymate® II robotic system, composed of a robotic arm, System V Controller, Hamilton syringe hand and an eight-channel pipetting hand, has been integrated with standard laboratory equipment, without significant modifications to form an integrated system capable of performing totally automated PCR experiments. Oligonucleotide primers and polymerase are pipetted with the Hamilton syringe into a 96-well microplate held at 4°C by the thermalcycler, thus reducing the waste and expense of disposable pipette tips. The cannula is washed with bleach and water to eliminate crossover contamination of primers. Target DNA is pipetted with the eight-channel pipetting hand functioning as a single-channel pipetter. An aliquot of air is drawn into the tip after the target DNA as a buffer to ensure no spillage that may cause DNA contamination. The mixtures are covered with an oil overlay with the eight-channel pipetter, disposing tips between additions to prevent crossover contamination. The program allows the operator to choose in any combination from 1–20 different primer sets and 1–96 different DNA samples.

Amplification in automated hot-start and conventional PCR experiments was at least as good as manual experiments. In addition, no contamination has been attributable to the robotic system. These automated experiments reduce labour by more than 90% and affords as much as a 90% reduction in the number of pipette tips used. Maximum system throughput is 400 samples per 24 hours. The system employs no special equipment and is a simple, efficient, labour-saving and cost-reducing molecular biological tool.

### Environmental A

### Automated laboratory procedure for isolation of pesticides from surface and ground water by solid phase extraction

*Mark Sandstrom, Kevin Fehlberg, Steven Zaugg and Steven Smith, USGS National Water Quality Laboratory, Arvada, CO*

A laboratory robotic system was developed for solid-phase extraction (SPE) of trace concentrations of a broad range of pesticides from large-volume (1 l) natural-water samples. Determination of pesticides in water involves many steps, with extraction typically the most lengthy and labor-intensive. Automation is one of the advantages of SPE compared to other extraction steps. An automated large-volume SPE system has been developed and tested using a custom-designed Zymate XP robotic system

(Zymark Corporation, Hopkinton, MA) that allows continuous and unattended processing of up to 25 water samples. The robotic system cleans and conditions the SPE cartridges, pumps samples through the cartridges to isolate the pesticide, dries the cartridge, elutes the adsorbed pesticides with solvent into test tubes, and cleans and rinses the sample lines between samples. A laboratory balance is used to gravimetrically document the initial sample weight and final sample weight processed through the SPE cartridge. The robotic system simultaneously processes samples in sets of five. Addition of the internal standard, evaporation of solvent, and analysis of the sample extract by gas chromatography mass spectrometry (GC/MS) operated in the selected-ion-monitoring mode are performed off-line.

Processing times for robotic samples (130 minutes per set of five samples) are longer than for manual samples because of the sequential drying of SPE cartridges used by the robotic system. Nevertheless, the robotic system potentially can increase sample throughput by 25% to 200 samples per week because it can be operated overnight. The robotic system can substantially improve laboratory productivity because personnel time required for sample preparation is reduced from 8 hours to 1 hour. Contamination and carryover in blank samples and poor recovery of low concentration (0.1 microgram per litre) quality-control samples were problems that were corrected during the development of the method.

### Extraction and enrichment of pesticides, metabolites and similar compounds by automated solid-phase-extraction and determination by gas chromatography and high performance liquid chromatography

*Claus Schlett, Gelsenwasser AG, Gelsenkirchen, Germany*

In the last few years the solid phase extraction for the determination of organic micro pollutants in water analysis has largely replaced the liquid/liquid extraction with solvents which are toxicologically precarious to environment. To obtain almost quantitative extractions, good reproducibilities and high precision, it is nevertheless necessary, to observe and follow strictly decisive parameters of analyses. With systems available to date for analysis, either self-constructions or also commercial products, working under defined conditions has not been possible. Besides, only an exact establishment and reproducibility of marginal conditions allows optimization of analytical steps. Unsatisfactory observance of intermediate steps resulted, especially in lower analytical ranges, in considerable deviations of results. With Zymark's AutoTrace, a unit of equipment for automated solid phase extraction is now available, which enables working under exactly defined parameters.

This analytical unit was tested in the central laboratory of Gelsenwasser AG for its applicability regarding extraction and concentration of a multitude of components in the lower analytical working range. The examination included pesticides, polychlorinated and polybrominated biphenyls, anilines, nitroaromates, polycyclic aromatic hydrocarbons, odor components and so on.

Compared to the usual extraction, optimization of equipment parameters and observance of frame conditions dependent on substances led to an evident increase of reproducibility and precision for most components. Besides, it became apparent, that a specified solid phase/water ratio, the drying time, the speed of concentration (Load Flow), the speed of elution (Elute Flow), as well as the addition of organic solvents to the water sample have a decisive influence on the quality of analyses.

The reaction time of an organic solvent with the solid phase material certainly does not play the leading role in conditioning and elution, but, nevertheless, has a perceptible influence on the quality of the analyses.

### Automated extraction and analysis of chlorinated compounds in water at 10 ng/l level using robotics and large volume on-column injection

*R. Tamilarasen, P. Morabito, Dow Chemical, Analytical Services, Midland, MI; A. Butt and P. Hazelwood, Dow Chemical, Sarnia, Ontario, Canada*

A laboratory robotic system was used to automate the method for the determination of 16 neutral chlorinated compounds in water. Samples are loaded into tared vials on the system followed by the addition of extraction solvent into the vials. The extraction is performed by shaking on a linear shaker followed by vortexing to break the emulsions which may be formed during the extraction procedure. The robotic table is coupled to a gas chromatography/mass spectrometry, GC/MS, equipped with a large volume on-column injector, LOCI. The extract is sipped through a 100 μl sample loop installed on the injection valve. The injection is made using retention gap and valve switching technology to vent greater than 90% of the solvent before the anlalytes desorb onto the analytical column. Instrument linearity, precision, and method detection limit have been determined and meet the requirements of the Ontario Ministry of Environment MISA program. A Design of Experiment approach was used to optimize the extraction conditions.

The automation reduces the amount of solvent required for the extraction by 80–90%. Significant savings in operator time will be realized at the current level of sampling. The use of GC/MSD provides highly defensible data with positive identification of the compounds. No significant degradation of the analytical column was noted after more than 2000 large volume injections.

### Pharmaceutical research and drug discovery

### Automation for high throughput screening and sample distribution using a tracked robotic system

*Derek J. Hook, Joe Y. Yacobucci and Jeffrey Guss, Bristol-Myers Squibb, Pharmaceutical Research Institute, Natural Products Research, Wallingford, CT*

During the authors' use of robotic systems for high throughput screening in the pharmaceutical industry, they have continued to optimize the integration of the

Zymate robotic system with commercially available laboratory instrumentation.

Their initial experience with ELISA systems made use primarily of components supplied by Zymark but with the use of a few items of commercial equipment such as the Bio-Tek and BioRad plate washers, which were reported on at ISLAR 87. As experience was gained with the robotic arm for material handling and less for functions such as liquid handling, plate washing and photometery, it was found that these can be performed more efficiently by dedicated commercially available instrumentation. The authors reported on the use of such a system at ISLAR 92 for the distribution of natural product extract samples for high throughput screening.

The major disadvantage of the system was the limited access by the Zymate robot to only one side of the Hamilton MPH 2200 liquid handler. Since that presentation a Zymate robot has been used on a linear track (similar to that described by Kanczewski at ISLR 92 for stability testing), to access fully the XYZ decks of Hamilton liquid handlers from the front, thus increasing utilization of the excellent liquid handling capabilities of the latter instrument.

The use of front access to commercially available and custom built equipment using a Zymate robot on a seven foot linear track was described. Procedures for use of the system for sample distribution and high throughput cell based cytotoxicity screening were discussed. Front access considerably simplifies the programming of the Zymate robot system, and the positioning of equipment in a non-radial configuration about the robot seems to improve the robustness of the robotic procedures.

### Automation of an antiviral screening using a Zymate laboratory robot

*Rudy Willebrords, Koen Andries, Alfons Vander Auwera and Roger Rosiers, Janssen Research Foundation, Beerse, Belgium*

Drug screening in the pharmaceutical industry usually employs biological assays to detect pharmacological activity. *In vitro* antiviral assays, generally based on the cytopathogenic reduction method, are microscopically examined. Recently, rapid and more sensitive *in vitro* procedures, based on spectrophotometrical assessment for viability of virus- and mock-infected cells were developed to evaluate antiviral agents via *in situ* reduction of a tetraxolium dye MTT.

The labour-intensive nature of different antiviral tests and large number of test compounds to be processed meant that the authors' laboratory was a feasible candidate for automation. The authors have developed a semi automated procedure for utilizing a Zymate Laboratory Automation System to screen antiviral compounds against different human viruses, for example rhino-, herpes simplex- and influenza viruses.

A procedure using this system was described which includes dissolving, aliquotting, assaying and spectrophotometrical measurements of test compounds. The whole application is performed in a specially constructed conditioned room (temperature and humidity). By moving the robot over rails on an X–Y axis, it has access to different parking places after well-defined incubation times (hours or days). The robot transports the plates from or to sterile work area (laminar flow hood) in order to add drugs and dye solutions or spectrophorometrical evaluations.

The automation of the screening system allows the authors to increase the capacity to test new substances in a rapid way, compatible with the manual assay, with lower costs, independent of normal work hours and less labour intensive.

### The DIVERSOMER approach: integration and automation of multiple, simultaneous organic synthesis on a solid support

*Shelia Hobbs De Witt, Charles J. Stankovic and Mel C. Schroeder, Parke-Davis Pharmaceutical Research Division of Warner-Lambert Company, Ann Arbor, MI*

The generation of chemical diversity by the simultaneous synthesis of 40 potential drug candidates has been achieved in the authors' laboratories. This innovative technology combines solid phase chemistry organic synthesis, miniaturization, robotics, and a unique apparatus for multiple, simultaneous synthesis to generate libraries of organic compounds (DIVERSOMERS).

A fully integrated and automated DIVERSOMER system requires exploitation of several discrete computational tools from the initial setup to submission of the final compounds for testing. The transfer of data and samples within the laboratory and between independent departments requires communication between operators, laboratoey instruments, computers, and robots.

The Tecan robot has been extensively developed and used for liquid sample handling and reaction monitoring for the DIVERSOMER project. Modifications to the Tecan robot, have enabled reaction monitoring by TLC analysis, injection or withdrawal of reagents through a gasket (within a controlled atmosphere), and sample preparation for final product analysis by NMR or MS.

Microsoft Excel spreadsheets have been extensively used for the tracking and manipulation of data. Using excel as a platform, electronic data transfer across a network has begun to integrate the multiple components of the system including instrument control, analysis of products (both within the laboratory and between other laboratories), and final compound submission.

### Development of an automated compounds dissolving laboratory in a pharmaceutical research centre

*Pierre B. Monnet, Frederic Carlier and Lionel Drugeault, Rhone-Poulenc Rorer, Vitry-Alfortville Research Center, Vitry-sur-Seine, France*

The mission of RPR Central Research is to ensure a constant flow of innovative products and new chemical entities that target major unmet human medical needs. Carrying out this mission means unceasing growth of the number of screening assays.

In 1991, an automated system called DAUPHIN was designed to deliver quickly and accurately the compounds to be assayed. This system induced the dissolving of compounds to become the new bottleneck of the screening line.

A second automated system called MISTRAL was designed to control the dissolving of compounds in order to place the proper solution at the researcher's disposal. This sytem allows the choice of the appropriate solvent (one of six solvents), as well as the required concentration, both targets being reached whatever the amount of compound. Moreover, the compound dissolution is checked by a turbidimeter, allowing the system to dilute the compound until the total dissolution.

The paper described the stand-alone module technology associated in the use of two Zymate® robots sharing an overlap area. This technology induces the system to control two intelligent storage devices, one turbidimeter with its automated dishwasher, two intelligent automated dissolving devices and the robots.

Furthermore, the ability to control directly the two robots and the peripheral devices by one computer (presented in the poster session) has allowed the development of a synchronous-multi-task software. This software controls all the devices in order to improve the actions scheduling as well as to synchronize a device's action with another.

Finally, the authors examined the advantages of this automated laboratory. This automation allows time saving by working during the night. The samples are at the researcher's disposal early in the morning.

MISTRAL has been working since March 1993.

### Environmental B

### Eight years of robotic PCB-in-oil preparation

*Richard C. Peck and Joseph J. Marinaccio, Northeast Utilities, Laboratory Services, Hartford, CT*

Over the past eight years the authors' laboratory has analysed over 100 000 oil samples for polychlorinated biphenyl (PCB) content. A Zymark robotic system customized to fit their preparation needs was used for sample preparation, followed by subsequent analysis with a gas chromatograph. Overall, the quality of PCB analysis showed significant improvements, while the risk of injury to laboratory personnel decreased and the manpower required to test a sample also decreased by about 80%.

Oil samples are precisely diluted with hexane solvent in scintillation vials using an analytical balance. The sample is then cleaned up using concentrated sulphuric acid followed by the transfer of the diluted sample to a 1 ml vial. A unique dispenser for 1 ml vials has added reliability to the system. Difficulties encountered and solved during the past eight years include the capping of oil scintillation vials, the capping of 1 ml vials, depending of solvent, and the transfer of liquids.

### Development of a new automated analytical method for pesticides in soil

*Guenter Bachlechner, Bayer AG, Crop Protection Development, Institute for Product Information and Residue Analysis, Bayerwerk, Germany*

The requirements for residue data in soil have changed during the last years. The limit of determination necessarily moved to extremely low values. In addition to this the analyses of soil are to be done with a high precision and reproducibility. This is why residue analysis in soil became very labour and cost intensive.

In an integrated approach, the most time-consuming steps and the sources of variation were identified. Different analytical techniques were tested to eliminate the time-consuming tasks (to reduce costs) and to reduce the sources of variations. As an example, the automated analytical method for imidacloprid in soil is presented.

Outline of the method:

Soil samples are extracted in a 'Soxtec-Hot-Extraction-Equipment' with boiling methanol. The extraction time is 60 minutes and the rinsing time is 30 minutes. The solvent is evaporated and the residue is cleaned-up by a laboratory robotic system with column chromatography on silica gel. The samples are dissolved in toluene, transferred to the silica gel column and impurities are removed from the column with toluene/ethyl acetate (7 + 3 parts by volume). The active ingredient is eluted with ethyl acetate, evaporated to dryness and dissolved in acetonitrile/water (1 + 1 parts by volume). The quantitation is done by high performance liquid chromatography with UV-detection at 270 nm.

The mean recoveries of the method, which were determined in the range of 0·0006 to 0·174 mg/kg, were 88·0% for imidacloprid with a standard deviation of 12·4% (relative standard deviation: 0·14). During analyses of samples of field dissipation studies considerably lower values for the standard deviation in the range of 1 to 4% were found.

The lower limit of the practical working range of the analytical method was 0·006 mg/kg for imidacloprid (limit of determination). The lower limit of the range of detectability for imidacloprid was 0·002 mg/kg (detection limit). Above this concentration a peak could be differentiated from signals of blank samples.

There is a potential to save costs and work in residue analysis. To profit from this, investment of money, manpower and experience is necessary. It was possible to reach both goals: to reduce costs and to enhance the analytical quality of the results by introducing new analytical techniques and optimizing the single steps of work and sample flow in the laboratory. This example of soil analysis shows that the investment of money and manpower can be returned within one year.

### Factors affecting the reliability of solid phase extraction methods in environmental samples

*Margaret Raisglid, University of Arizona, Tucson, AZ*

The use of solid phase extraction (SPE) in environmental analysis is one of the fastest growing areas in analytical chemistry. The challenge in developing SPE procedures is to selectively concentrate the analytes of interest while maximizing their recovery.

Some factors influencing the selectivity and recovery of analytes include the sample loading and elution rates, choice of sorbent materials, as well as selection and volume of elution solvents. The influence of sample pH and the addition of salts and solvents to samples prior to loading on to the SPE column can have a significant impact on analyte receovery. For applications where analyte elution is with a water immiscible solvent (for example EPA Method 525.1), column drying techniques between solvent transitions can be critical, particularly with respect to choice of drying gas and optimization of drying time.

Failure to consider these various aspects in SPE method development can result in non-robust procedures, lengthy development times and excessive costs.

The influence of varying several of these parameters on analyte recovery were discussed.

### Automated extractions of ground waters for polynuclear aromatics on the AutoTrace workstation

*Richard A. Kern and Krystyna Z. Czyzo, Midwest Analytical Services, Detroit, MI; and David A. Williams, Zymark Corporation, Hopkinton, MA*

There are some reasons for converting from Liquid/Liquid Extraction (LLE) sample preparation methodologies (EPA 3510) to Solid Phase Extraction. Traditional, LLE is labour intensive and produces large quantities of waste solvents such as methylene chloride. SPE procedures are less complicated than LLE. This reduces set-up and extraction time, training time, and the amount of glassware used. SPE procedures use less organic solvent than LLE procedures. This decreases the cost of analysis, technician exposure to chemicals and the cost of waste disposal.

Vacuum manifold SPE requires a level of technique to ensure quality data. Performing the conditioning properly, loading the sample at a constant rate, and eluting the sample consistently require time and attention to technique which is still costly and demands a high level of skill.

AutoTrace automates the tasks of SPE sample preparation. Operator bias is eliminated and the amount of time spent performing sample preparation is reduced by 80%.

### Advanced topics

### Optimization of carry-over

*I. B. Anderson and Povl Nilsson, Novo-Nordisk A/S, Hagendornsvej 1, Gentofte, Denmark*

Carry-over of material is an inevitable part of sample transfer when a stainless steel probe coated with Teflon is used for aspiration and dispensing purposes. A rule of thumb states that the wash volume of the probe ought to exceed ten times the pipetting volume, and shall not be less than 500 µl. It is of importance that the wash volume is sufficient to insure that the carry-over is below a certain (acceptable) level. That can always be achieved by increasing the wash volume. Use of too much wash volume means on the other hand waste of wash reagent, time and money.

Test systems have been developed, so that the carry-over can be measured either by photometry or by radiactive measurements in set-up which equal the analytical set-ups. The wash volume which insure a carry-over below an acceptable limit can be determined directly by means of the tests. Carry-over values less than 1% can be determined directly by means of the tests. Carry-over values less than 1% can be determined directly by photometry. Radioactive measurements can determine carry-over in the $10^{-6}$ range. The time for making a determination of the carry-over and of the necessary wash volume is nearly one hour when using photometry, and over-night for radioactive measurements. The test systems were described and results presented.

### Dispensing corrosive reagents with a Zymark system

*William R. Kew, Express Analytic, 3131 Woodcrest Drive, Downers Grove, IL 60515*

A Zymark XP robotic system designed to determine fatty acids in food performs sample preparation steps prior to GC analysis. The saponification and derivatization steps require $BF_3$/MeOH and NaOH/MeOH, very corrosive reagents. The initial design used MSL glass syringes. $BF_3$/MeOH pitted the metal syringe plunger and etched the glass barrel. NaOH/MeOH left deposits on the syringe walls that eventually caused seizure. Weekly replacement was necessary, expensive and highly unacceptable.

A method was designed which allows reagent dispensing without syringe degradation. This method uses three MLS valves, one syringe, and a cannyla with a holding loop. Custom programming loads the chosen reagent into the holding loop using an air gap to prevent dilution. This system has been operating successfully for six months with no maintenance due to reagents. Intermediate designs were described and the advantages and disadvantages of each were discussed.

### Experimental control system for the robotic material processing system

*Michael E. Dobbs, ERIM, Space Engineering and Material Science Department, and Space Automation and Robotics Center, Ann Arbor, MI*

The Experiment Control System significantly lowers program risk and cost, while providing the microgravity science community with state-of-the-art research capabilities. The ERIM ECS enables the scientific user to have direct control of their experiment. This access significantly enhances their capabilities to perform the unknown. Additionally, the ECS is capable of production-scale automation that is essential to attracting the untapped microgravity 'enabled' materials consumer base.

The ECS is large independent of specific experimental or production processes and associated carrier interfaces. It accomplishes this by rigorous adherence to modular architecture and reusable elements. The ERIM approach achieves previously unobtainable low development and life-cycle costs. The reduction of on-orbit research and production costs are essential to attract large numbers of industrial users. A large user base creates a competitive space automation and robotics supplier industry.

The high performance and low cost of the ECS system is only possible through ERIM's utilization of innovative commercial technology. The ECS is built around two off-the-shelf components: (1) the Zymate Laboratory Automation System developed by Zymark Corporation; and (2) the Spacecraft Command Language co-developed by the Naval Research Laboratory and Interface and Control Systems Incorporated.

The ECS's modular architecture and reusable components meet new science, manufacturing and mission requirements with minimal development time and cost. The innovative technologies in ECS are uniquely available to ERIM through co-operative commercial development and license agreements between private industry and ERIM.

### Development of a customized laboratory information management system (LIMS) for data acquisition and analysis for integrated high throughput drug discovery

*Ron E. Delmendo and Nick E. Wharf, Natural Products Discovery Group, Panlabs, Inc., Bothell, WA*

Biological screening of samples for the discovery of novel pharmaceuticals has witnessed several significant advances within the last decade. Advances in robotics and laboratory automation, the adoption of the 96-well plate format as the standard for testing, and an increased understanding of the physiological processes open for possible therapeutic intervention has taken place throughout all of scientific research. Nowhere is this more apparent than in drug discovery laboratories where high throughput screening has evolved from being a buzz-word to a job description. A side-effect of high throughput screening is the generation of reams of data which must be acquired, analysed, sorted and stored.

The authors have designed a customized Laboratory Information Management System (LIMS) for their Natural Products Discovery Laboratory. This LIMS was written in Borland C++, Visual Basic for Windows and Paradox for Windows PAL programming language. Program modules for data acquisition from each of the authors' laboratory counters were designed independently and linked to analysis software. This 'building block' approach provides added flexibility to the system when dealing with multiple data output interfaces.. The LIMS software is run of a Novel Local Area Network (LAN) to provide linkage between laboratory counters, user workstations and database storage. The system takes advantage of the Windows environment to allow multi-tasking and multiple user performance.

### Automating pharmaceutical methods

### Fully automated, dissolution profile testing in a microsoft-windows environment

*P. Waters, D Mullen and B. Harward, Glaxo Research Institute, Research Triangle Park, NC*

Dissolution testing in the Analytical Chemistry department at Glaxo Research Institute often requires multiple data points in order to construct a dissolution profile. On-line sample UV absorbance measurements are taken, typically at five minute intervals, over a period of 45 minutes. A standard Zymark dissolution robot system had been previously developed to perform these tests. However, the design of the sampling system resulted in overall run times (including vessel setup and cleanup) in cess of five hours to complete testing for six samples. The authors sought to improve the efficiency of the Zymark system, such that the required sampling intervals would no longer define the rate-limiting steps of the test.

The resulting PC-based system uses the Zymark robot for vessel preparation, sample introduction, and vessel cleanup. The Hewlett-Packard 8452A Diode-Array spectrophotometer with the Multi-Cell Transport Mechanism option is used to provide a closed-loop configuration for simultaneously withdrawing samples and measuring UV absorbance. In addition, a Microsoft (MS)-Windows Visual Basic program was developed to provide top-level control of the entire system; to provide a user interface; to activate the HP8452A dissolution software; and to control bi-directional communication between PC and the Zymark system. Using the System V controller Autoload function, the Zymark system runs entirely in background; therefore all user interaction is maintained within the MS-Windows environment.

Sampling intervals as frequent as 3 minutes are now possible, while the overall run time (including setup and cleanup) is dependent only on the total sampling time required.

### The best of both worlds: analytical results from a dual-cream and tablet PyRobotic system

*Allan Greenberg, Richard Young and Phillip A. Lane, R. W. Johnson Pharmaceutical Research Institute, Raritan, NJ*

A dual PyRobotic system was described and shown to be capable of assaying both a viscous cream, using the Viscous Liquid Hand, and small low dosage oral contraceptive tablets. The logic used to program the robot with respect to including both standard and sample preparation in the case of the cream and using only sample preparation in the case of the oral contraceptive tablet was discussed. Analytical data were shown comparing the results and demonstrating the equivalency of manual and robotic sample preparation of cream and oral contraceptive samples. The assay reproducibility and sample throughput were presented for both cases. Also discussed were the security precuations implemented on the robotic systems and PCs to prevent accidental changes in the programming code or any function that might be detrimental to the robot from completing the assigned task.

### An automated content uniformity application: from Zymate Tablet Processing Workstation to BenchMate

*Dorothy E. Martynuk, Arjun G. Sheth, Daniel Zuucarello, Alexander D. D'Addio, Henry Mortko and Kevin J. Halloran*, Wallace Laboratories, Cranbury, NJ*

An automated content uniformity application for the HPLC analysis of pharmaceutical tablets has been developed and validated using a Tablet Processing Workstation (TPW) together with a BenchMate Workstation. The first part of the application consisted of the development and validation of a Zymate TPW method for the homogenization of tablets and the extraction of the drug component of interest from the tablet matrix. The second part of the application was the development and validation of a BenchMate Workstation procedure for the filtration and injection of sample solutions onto a HPLC system.

Validations of the TPW and the BenchMate were conducted independently. Separate validations provided us with the flexibility to schedule independent workstation use. This rationale permitted the full utilization of each workstation for handling samples in several combinations of preparation schemes. In the first scheme, the TPW is configured for automated extraction. Samples prepared by the TPW can follow one of two post extraction routes. An analyst can manually filter the solutions for off-line HPLC analysis, or a BenchMate procedure can be employed to filter the solutions for on-line injection onto a HPLC system. In the second scheme, the BenchMate Workstation can independently filter and inject sample solutions prepared from different sources. It can accept manually extracted sample solutions or sample solutions prepared by the TPW. In the final scheme, the two workstations can operate together as a single automated system.

This paper discussed the validations in greater details with respect to recovery, residual drug carry-over, injection reproducibility, filter binding and comparisons of manual with automated procedures.

* Present address: Knoll Pharmaceutical, Whippany, NJ.

### An automated system for the quantitative collection and determination of doses delivered through the valves of metered aerosol products

*T. A. Finley, N. A. Landes, Rhone-Poulenc Rorer Pharmaceuticals, Fort Washington, PA; R. M. Fuchs and A. J. Zepka, InnovaSystems, Inc., Merchantville, NJ*

The collection and analysis of doses from metered aerosol products has been found to be a tedious, labour-intensive procedure. Furthermore, analyst-to-analyst variation in the actuating and collecting of samples from the canisters has been shown to be a significant, albeit difficult to quantitate, variable in such determinations.

Automation of the testing process conserves analyst time and eliminates all the variables associated with manual testing, thereby producing more consistent results.

Five major challenges were overcome in the development of this system:

(1) An actuator mechanism capable of both wasting and collecting metered doses had to be developed. In addition, this actuator needed to shake the canisters and prevent waste actuations from entering the laboratory environment without the need for a dedicated fume hood.
(2) A method to accommodate multiple waste/collection actuation protocols as well as differing canister sizes had to be designed. Also, the option to weigh the canisters at various stages of the process was necessary.
(3) Cost had to be contained within a relatively tight budget and return on investment had to be realized. This was done by recycling existing software to this application as appropriate.
(4) Finally, data storage and retrieval to and from an existing, secured automated data system was required.
(5) In order to provide redundancy, the system had to be designed to operate with one or more actuators.

The system consists of a Zymate II Plus Robot, Compaq 386/16 PC, 2 custom-built canister actuators, an analytical balance, a UV-visible spectrophotometer, and a solvent pump and fill station, as well as racks for the canisters, beakers, and standards.

All activities of the system are co-ordinated and controlled by the PC under a scheduling prioritization algorithm. The movements of robot are executed with the System V controller in response to value parameters passed to it by the PC. The actuators are pneumatically operated, with the positions of the pistons detected by magnetic sensors and driven by the PC. In order to meet the throughput demands, operations execute simultaneously whenever required.

This paper discussed details of the design, implementation, and operation of the system.

### Implementing a Zymate II robot in the QC environment

*Jon P. Sadowitz, Danbury Pharmacal, Carmel, NY*

Content uniformity (CU) and assay testing for drug products in the pharmaceutical industry entails a great expense of time, money and workforce. It is very important that these tests results are accurate and precise. These criteria along with the increasing volume of samples through the QC lab, prompted Danbury Pharmacal, a division of Schein Pharmaceutical to implement a Zymate II Robotic System for finished product UV analysis. The implementation of the system for routine on-line analysis in the QC lab, has saved Danbury Pharmacal time, money, and provided better utilization of the workforce.

The current processing time for assay and content uniformity via manual method, is approximately $4\frac{1}{2}$ h versus approximately 45 min of analyst intervention time for the robotic method. This not only is a time saver for the analyst, but shortens the turn around time of analytical testing from two days to one day, by allowing the analyst to perform tests that otherwise he would not have time to do.

The accuracy and precision of robotic analysis versus manual method analysis is equally impressive. The validation statistics of method equivalency—manual versus automated for 100 mg Trazadone, show the statistics of the automated method to the manual method. The average assay of 50 samples over 5 lots for the manual method was 98.62 with an RSD of 1.85. The average for the same lots for the automated method was 98.93, with an RSD of 1.04. The obvious advantage is that the robot does not possess analyst to analyst variation.

To implement this system into the QC lab to perform on a routine basis, a strategy first had to be developed The strategy covered the areas of Product selection, Benchtop Validation, Method Validation, Program Software Development and Validation, QC analyst training, and other areas that encompass bringing online a robot in the QC environment.

### Validation of the BenchMate for dilutions

*Robert J. Engerer, John C. Egoville and Mike C. Penrose, Department of Analytical and Physical Chemistry, Rhone-Poulenc Rorer Central Research, Collegeville, PA*

A manual dilution procedure which involves a consecutive five-step dilution to produce two final concentrations has been automated using the BenchMate. The task for making manual dilutions requires a chemist trained to avoid the 'pitfalls' encountered with dilution methods. Because fractions of the standard volumes are required, 'standard glassware' is not used. When the strength of the medication changes, the chemist has to be alert to change dilution ratios and the lengthy manual procedure. The BenchMate provides the chemists with a versatile and accurate tool for making quick method changes and completing the daily dilutions.

A BenchMate three-tube dilution procedure reduces the five-step manual dilution procedure to a three-step procedure. Three BenchMate procedures have been tested: the transfer, dilution, and dilution–transfer programs. The advantages and disadvantages of each method were presented.

A Lotus macro program has been developed to determine the dilution ratios and the weight per gramme for each step in the BenchMate procedure. This makes determining the BenchMate parameters for new formulation strengths quick and easy. A second Lotus macro program was developed to read step and labels and weight data off the BenchMate disc and calculate final concentrations for a total of 50 samples. The advantages of automated dilutions by the BenchMate is not only in terms of labour savings but also in terms of quality assurance. All dilutions are verified by a printed record of solution weights and volumentric concentrations before samples are submitted for testing.

Samples for all strengths were diluted by the BenchMate and analysed by atomic absorption to verify all dilution ratios, cannula transfers, and mixing steps were accurately performed. Concentrations above and below the desired final concentrations were also produced to bracket the samples.

### Custom automation

### Automated high temperature polymer dissolution system for gel permeation chromatography

*Paul Morabito, Dan Duke, Dow Chemical Company, Midland, MI; Drew Poche and Ray Brown, Dow Chemical Company, Plaquemine, LA*

Manual preparation of polymer samples for characterization by solution methods such as gel permeation chromatography (GPC) is a time consuming, labour intensive, and redundant task. A typical manual sample preparation involves adding 1,2,4-trichlorobenzene to the polymer sample, heating the mixture to 160°C, filtering and transferring the hot solution to an autosampler vial. This exposes the analyst to both hot surfaces and solvent vapours.

An automated system to prepare samples for high temperature GPC analysis was developed for the Louisiana Analytical Division Polymer Characterization and Fundamentals Group. The system is based on Zymark Laboratory Robotic System and custom hardware peripherals developed at Dow Chemical. The system performs all the steps required to prepare samples for high temperature GPC analysis and transfers the hot solution to a GPC autosampler vial for the Water's 150C chromatograph. The automation challenges, system performance, and future direction were presented.

### Development of a high throughput high quality DNA sequencing sample preparation robotic system

*A. R. Watson, N. Smaldon, K. Karunaratne and R. Lucke, Sanger Centre, Cambridge, UK*

The Sanger Centre is a large DNA sequencing centre. Sequencing projects include the *Caenohabditis elegans* project, human genome, and yeast genome sequencing projects. A multi-purpose machine that carries out a large number of the sample processing requirements of the projects has been developed. The system consists of a large (1·5 m × 0·5 m × 0·2 m) 3-axis pipetting robot. Clean disposable tips can be picked up by the robot from a bowl-feeder which sorts and orients up to 3000 yellow tips, input in bulk. Four temperature cycling blocks can be accessed by the robot. Also a video camera system allows for vision-guided processes. The system carries out the tasks of:

(1) Plaque picking—M13 plaques are automatically picked from agar plates into Eppendorf tubes for growth. This is achieved by digitizing images of the plates using a video camera and computer interface card. Analysis then proceeds automatically to identify plaque co-ordinates. These co-ordinates are then used by the robotic part of the system go guide the robotic head to the plaques.
(2) Template preparation—M13 supernatant is transferred from Eppendorf tubes. A novel biochemical technique using magnetic beads is then used by the robotic system to purify the DNA.
(3) Preparation of glycerol storage stocks—Samples from the bottom of each Eppendorf tube are mixed, with glycerol, to microtitre plates for storage at −70°C.
(4) Sequencing reactions—Cycle sequencing reactions are performed using the purified DNA. Samples are then pooled ready for manual precipitation and loading on to the sequencing gels.

The systems developed can each process 600 samples per day through all the steps listed and are currently in use on all the sequencing projects outlined above.

### A solid reagent handling robot

*Thomas R. Smith, James W. Streetman and John A. Lopez, Jr., Shell Development Co., Houston, TX*

The robotic handling of solid reagents poses a number of challenges. When the reagents are air and moisture sensitive, the problem of designing robotic compatible dispensers is complicated even further. A custom robotic system designed to handle just such reagents was presented. The system is used to prepare the water and the carbon dioxide absorbers that are used by a robotic carbon/hydrogen determination system implemented several years ago. Preparing these absorbers manually is a tedious, time-consuming task. The absorbers consist of several layers of different reagents that must be added in the proper quantities to obtain correct carbon/hydrogen results. The absorber packing robot system is designed to handle all aspects of the absorber preparation including gravimetric validation of material dispensed and leak testing of the completed absorbers, thus freeing personnel from a boring task and assuring a steady supply of consistently prepared absorbers.. The system includes custom workstations that dispense fixed quantities of air sensitive reagents, dispense and mix multiple reagents in a predetermined ratio, place glass wool plugs in the absorbers (necessary to prevent reagent loss), and cap the absorbers when completed.

### Application of laboratory automation to food analysis

*W. Jeffrey Hurst and Robert A. Martin, Jr., Analytical Research, Hershey Food Technical Center, Hershey, PA*

In the analysis of food and food components, laboratory automation has played an important role ranging from the introduction of autosamplers for chromatography to the development of various turnkey assays using laboratory robotics. This presentation outlined some selected uses of laboratory automation in food analysis, including sample preparation for HPLC and GC, laboratory robots serving as autosamplers for various types of instrumentation including NMR and NIR, turnkey systems for fat analysis, workstations that perform dedicated and specific analyses and finally automation in SPE that can be used for automated fat analysis.

### Automated approaches to polymer solution characterization

*Arthur Wilde, Goodyear Tire & Rubber Company, Akron, OH*

The Goodyear Tire & Rubber Company is well known as a major manufacturer of tires and engineered rubber products. However, Goodyear is also both a major producer and consumer for synthetic polymers, and as such has come to rely on high quality polymer characterization by spectroscopic and chromatographic techniques to provide valuable information on polymer properties. However, these methods rely on the polymers being soluble in solvents, and the precise concentrations of these solutions must be known.

Since 1984, the Polymer Characterization Section of Goodyear's Corporate Research has used increasingly more sophisticated robotics methods to support polymer analyses and polymerization research. Beginning with a single arm Zymate I system doing very simple sample preparations for GPC, this group now uses several customized robotics applications to support all areas of solution characterization.

Conciding with the advent of Total Quality Culture (TQC) at Goodyear, a custom method to measure polymer solubility was designed. The TQC method calls for incremental and verifiable improvements in quality. Using TQC principles, a method for measuring polymer solubility, which has the required accuracy and precision to support the chromatographic analysis, has been developed for automated systems.

This paper discussed the implementation of lab robotics to support polymer characterization at Goodyear. Simultaneous improvements in data quality, data output rate, and equipment utilization were documented, and the successful transfer of this technology from the Research laboratory to Development and manufacturing Quality Assurance laboratories was shown.

### Automated space production experimenters network (ASPEN)

*Michael E. Dobbs, ERIM, Space Engineering and Materials Science Department & Space Automation and Robotics Center, Ann Arbor, MI*

The Space Station presents opportunities for space-based research over a longer time span. Current space-based experiments have relied on human interaction during their processes. A high level of automation will be required, however, to expand on-orbit accessibility, productivity and efficiency. The Automated Space Production Experimenters Network (ASPEN), utilizing proven laboratory automation and robotic techniques, will provide researchers with an easy-to-use, multi-purpose, cost-effective solution to access to space. A 100-fold increase in users is anticipated.

ASPEN successfully addresses the issues raised by the scientific, astronautic, industrial, and financial communities in the report entitled *Space Station Freedom Automation and Robotics, An Assessment of the Potential for Increased Productivity, March 1990*. The ASPEN program will develop and demonstrate (1) a multi-disciplinary life and material sciences facility by integrating state-of-the-art, off-the-shelf automation and robotics technology; (2) improved human factors; (3) low-cost infrastructure for private/public research/industrial users; (4) a business plan for a leased/rented facility; and (5) can be the first flight demonstration meeting the goals of the NASA EXPRESS program as a common experimental apparatus.

In addition, ASPEN could automate processes identified in the *Life Sciences Hardware Baseline, Rev. 1* report. The ASPEN does, self contained experiment scheduling and error recovery using Spacecraft Command Language; automated sample processing and analysis using Zymate Laboratory Automated System–System V controller and EasyLab language; 6-DOF robot and XP controller. ASPEN has 300 standard procedures and 2000 installations. ASPEN can be equipped with many accessories and instruments for automatic assays, image processing, PCG, microbial, biology, cell development, medical sample (urine, blood) analysis and HPLC.

Experimental costs can be reduced to below 50 000. This is significant to NASA as the means to enable a larger user base (Spacelab, SpaceHab, the Space Station, etc.) and other agencies (such as NSF, NIH and DOE) that fund experiments; thus obtaining private sector capital, and fulfilling the national need to fill the educational pipeline with students in science and technology.

### Plenary

### Laboratory robotics—poised for the 90s

*Francis H. Zenie and James N. Little, Zymark Corporation, Hopkinton, MA*

As laboratory automation enters its third decade, laboratory robotics is increasing in scope from automated sample preparation to sample automation. Sample automation includes all sample handling and preparation steps prior to instrumental analysis and the integration of sample data with analytical data.

Effective sample automation requires continuous technology advancements, new management approaches within laboratories and innovative relationships between using laboratories and vendor organizations. Automation must lead to application and business solutions where systems integration and value-added services are as important as excellent technology.

In today's competitive and highly regulated economy, laboratory automation must become a tool to help valuable people become even more effective.

### Posters

### Automation of an ELISA for detection of antibody to two antigens and data processing of vaccine clinical trial samples

*J. Patrick McCurley, Eric Hall, Mark Westley, Rick Najarian and Rose Sekulovich, Chiron Corporation, Emeryville, CA*

Au automated enzyme-linked immunosorbent assay (ELISA) has been developed for processing samples obtained from phase II/III vaccine trials. The assay utilizes a Zymark XP robotic system with microplate technology to analyze the amount of antibody developed against the two vaccine antigens (surface glycoproteins from Herpes Simplex Virus Type 2). Twenty-four samples are assayed on duplicate plates for antibody against two antigens in a five hour period. The system has a capacity of 96 samples in 4 cycles without operator intervention.

Bar-code labels with a unique identification for the trial, subject and visit are applied to serum samples collected at the clinical sites. The assay operator scans the label and places the septum stoppered tube into a custom temperature-controlled rack which is maintained at 2–5°C. The robot performs all operations in the assay including sample transfer, serial dilutions, preparation of conjugate and substrate solutions and plate reading. The raw data from the plate reader is captured on a SUN Sparc workstation. After all the plates in a run have been read the data is automatically transfered to a SUN 670 which calculates the raw and normalized titres, averages duplicates, spools hard copies to the printer and creates a temporary database which matches sample I.D. with results and flags any samples which are outside established standard criteria. The operator evaluates the results and transfers the acceptable data to a permanent clinical database.

Several equipment and program modifications were made to the original system including solution dispensing methods, a 37°C incubator door and object sensors.

### A dedicated workstation for delivering extracted fermentation broths into microtitre trays

*L. M. Ford, H. A. Boll, D. M. Bowden, J. M. Winnefeld, J. L. Woodrum and O. W. Godfrey, Lilly Research Laboratories, Lilly Corporate Center, Natural Products Research, Indianapolis, IN*

In discovery research, the constantly evolving need for automation that is both efficient and accurate has been the impetus for the design of a streamlined system for transferring fermentation broths into microtitre trays. This system requires less time and is more accurate than the system previously described by the authors (ISLAR '91). The workstation consists of a 96 channel peristaltic pump fitted with 12 Ismatec Pump Heads. Each pump head contains 8 rollers and 8 tube cartridges. The pump heads are geometrically centered above the fermentation module and are chained 4 abreast on 3 drive shafts. These drive shafts are rotated by a continuous double sided notch belt which is controlled by a stepping motor. Another stepping motor raises and lowers the dispensing head. Both stepping motors are controlled by a basic indexer driver. The various operations consisting of fill, dispense, purge and rinse are written with a Premiere Innovations lap top computer and then downloaded onto the indexer drive controller.

### An integrated system for high volume microplate ELISA processing

*Kirk Andree, The Hamilton Company, Reno, Nevada*

Microtitre plate based enzyme immunoassays are widely used in the clinical reference labs and more recently in drug discovery efforts based on mass screening. The need for a cost-effective and time saving laboratory automation with reproducible test conditions has resulted in a new Hamilton instrument, the Microlab FAME, or 'fully automated microplate ELISA' system. The design approach for this automated system was described.

### High throughput 96- and 384-well microplate pipetting

*Christopher Shumate, The Hamilton Company, Reno, Nevada*

The microplate format has become increasingly popular as a platform for procedures outside of the traditional clinical reference lab. The higher density 384-well plates allow efficient storage of large chemical libraries. A Microlab 2200 robotic pipettor (Hamilton Company) was equipped with a Multiple Probe Head (MPH) option holding eight independent fluid lumens. Their 9 mm spacing allows transfer from 96 well plates into the higher density 384 well plates through an interleaving pattern. The instrument can be used for reagent dispensing, master plate duplication, and gridding onto membranes for hybridization studies. A low volume version allows the transfer of as little as 100 nanolitres. An example of these procedures was presented with timing, accuracy and statistical variance highlighted.

### Implementation of a high speed pipetting station with the Zymate Laboratory Automation System

*Janice Skuse, Donna Phipps, Sally Quataert and Dace Madore, Lederle-Praxis Biologicals, W. Henrietta, NY*

The new Zymate Laboratory Automation System including a high-speed pipetting station is a prototype system designed to meet the future needs of Lederle-Praxis Biologicals for Enzyme-Linked Immunosorbent Assays (ELISA). This system was carefully designed to meet current needs to fulfill requirements for future applications. The authors' goal is to completely automate all aspects of the ELISA performance within 24 hours.

In order to meet strict timing considerations, Zymark developed and implemented a high-speed pipetting station for the Zymate System. All pipetting is handled by a separate station of the main robotic system. This station consists of an independently programmed robotic arm, a two tier platform which contains locators for two boxes of tips (96 well format), and two microtitre plates, four troughs fed by Master Lab Station dispenser, a single-channel pipette tip station, a 12-channel pipette, and a waste removal facility.

The main robotic arm shares tasks with the pipetting station thereby increasing sample processing efficiency. The high-speed pipetting station performs all predilutions and dilutions of unknown samples, controls and standard, allowing decreased set-up time for the technicians executing the ELISA. Use of this custom manufactured station provides greater sample throughput, faster sample turnaround time, and will allow precious samples to be put away before the end of the work-day.

Total automation of the ELISA offers many benefits such as freeing individuals from repetitive labour intensive work, while providing more consistent results with higher sample throughput. Multitasking by the main robotic arm allows multiple functions to be accomplished at the same time thus speeding sample processing. Implementation of the high-speed pipetting station not only allows increased sample throughput in the 24-hour time restriction, but also allows us to accomplish complete automation of the ELISA procedure.

### Robot pipettes in 512-well plate—work in progress

*Kevin Hennessy, Bill Mordan and John Shigeura, Applied Biosystems, Foster City, CA*

To demonstrate the feasibility of performing DNA sequencing reactions in a high-density plate, a reaction plate containing 512 wells has been constructed. Using a Catalyst robot, standard Taq cycle-sequencing reactions of single-stranded DNA have been performed. The goal of this effort was to quadruple the current batch size of the Catalyst and thereby increase the instrument's throughput.

### A fully automated screening assay for $cPLA_2$ inhibitors

*Karen Rigat, Maureen Laney, Chuck Villarrubia, Sophie Chiu, Randal Schatzman, Syntex Discovery Research, Palo Alto, CA; Michelle Palmer and Jasbir Seehra, Genetics Institute, Inc., Small Molecule Drug Discovery, Cambridge, MA 02140*

A fully automated, *in vitro* screening assay was developed to test compounds for inhibition of cytosolic phospholipase $A_2$ ($cPLA_2$), which plays an integral role in the inflammatory process. This assay is being used to screen libraries of compounds for novel anti-inflammatory agents. The system incorporates a Zymate XP microplate system for assay preparation, quantitation of prroduct formed during the reaction by a Packard A-500 Series continuous flow scintillation analyzer, and statistical analysis and report generation by a menu-drive SAS/AF program.

The enzyme reaction monitored in this *in vitro* assay measures the hydrolysis of a radiolabeled substrate catalyzed by $cPLA_2$. The amount of labeled product formed during the reaction is a direct indication of the enzyme activity present. The assay is performed in disposable 96-well microtitre plates.

A single personal computer allows operator interaction with both the Zymate controller and the Packard A-500 scintillation analyzer. The detector software in Windows identifies peaks of radiolabeled product and determines the area of each peak. The statistical analysis system was designed to correlate the concentration of compound in each well with the corresponding peak area. The estimate of $IC_{50}$ and its 95% confidence interval are obtained from the dose response curve fitted using a nonlinear modelling program in SAS. The $IC_{50}$ represents the concentration of compound at which the enzyme reaction reaches half its maximum response.

This system is capable of running 10 assay plates in each cycle of operation and has been used to test over 500 compounds.

### Automation of biological assays using a gantry robot and Tecan liquid handling systems

*Richard F. Hart, Mark W. Doring, Robert W. Bryant, David Pechter and David Kenyon, Microbial Products & R&D Engineering, Schering-Plough Research Institute, Kenilworth, NJ*

The first step in the drug discovery process involves identification of novel compounds that elicit therapeutically relevant biological responses. The concomitant requirement for identification of 'lead' compounds in all therapeutic areas included in a drug discovery program requires labor-intensive evaluation of numerous samples in a battery of therapy targeted biological assays. Automation of selected laboratory operations can facilitate 'lead' identification by permitting a higher rate of sample evaluation in a broader assay battery using fixed manpower resources. To accelerate the identification of 'lead' compounds, Schering-Plough Research Institute has developed an automated system that allows unattended operation of 3 Tecan RSP 5052 liquid handling systems using a gantry robot. The high payload, 4 axis robot addresses a 16 × 6 foot workcell using a gripper that permits on-line changing of end effectors. By changing end effectors the robot can handle individual 96-well plates, plate lids or the 13 × 14 inch pallets that are used to move sets of related labware between workstations. Reagents and plates are manually loaded onto pallets which are stored on sliding shelves that permit accurate positioning for robotic handling. Provision for refrigerated storage or incubation of biologicals and reagents is provided by a Forma $CO_2$ incubator and refrigerator each fitted with automated door opener to facilitate robot access. Workcell operation is scheduled by laboratory personnel using a spreadsheet interface. Task scheduling software, which runs on a Compag 486 in a multitasking environment using DOS/Desqview, coordinates and controls operation of all required workcell components according to the assay schedule provided. Liquid handling protocols developed and programmed by laboratory personnel using the Tecan Integrator programming language are initiated by the task scheduler once placement of all required labware by the robot is confirmed. Upon completion, pallets are returned to the storage area for timed incubations or removal for off-line processing.

### Design and validation of an automated ELISA for the measurement of IgG antibodies to Bordetella pertussis

*Boris Feld, Kelli A. Perri and J. R. Mezzatesta, Lederle-Praxis Biologicals, Pearl River, NY*

There is much effort currently to develop new vaccines against Bordetella pertussis (whooping cough) which will require large scale clinical studies and the analysis of hundreds of human serum samples.

A laboratory robotic microplate assay system has been utilized to quantitate levels of IgG antibodies to Bordetella pertussis in human serum by enzyme-linked immunosorbent assay (ELISA). All steps of this assay including washing, BSA blocking of antigen-coated plates, addition of samples, enzyme-labelled antibodies and substrate as well as reading of optical density were performed using Zymark robotic system (Zymate II robot).

Data reduction utilizes a parallel-line bioassay method in which the slope of the serial dilution curve is critical. During validation it was found that the slopes of the manual and robotic titration curves were significantly different. The cause of these differences was determined to be associated with the time taken by the robot to perform the serial dilutions in the assay microtitre plate. Several modifications of this step were performed to avoid the discrepancy.

Dilutions prepared in Beckman deepwell plates and transferred to assay plates resulted in better agreement between robotic and manual methods than that seen when dilutions were prepared directly in the assay plate. Also it gave the authors the ability to run 24 plates overnight (2 batches, 12 plates/batch) instead of 12 plates (2 batches, 6 plates/batch).

### Ethanol assay by GC gel matrix using the BenchMate Workstation

*William A. Maxwell, Sterling Health, R & D Center, Princeton, NJ*

The Zymark BenchMate II Workstation was used to transfer an ethanol gas chromatography assay in a gel matrix from a completely manual sample preparation to sample dilution and mixing provided by the robotic instrument. In doing so, a considerable time savings was realized by allowing the robot to automatically weigh the sampled delivered to the sample tube, calculate the amount of diluent required for a precise and reproducible dilution, and mix (via vortexing) into a homogeneous solution. The samples are subsequently transferred to a gas chromatograph for ethanol analysis. Previously, the manual method required several cumbersome labour-intensive tasks, such as weighing out the gel samples into volumetric flasks and performing serial dilutions. The automated method has been shown that it is statistically equal to the manual method with regard to % ethanol equivalent and also provides better precision than the manual method. The automated method is now used on a routine basis.

### Automated mycotoxin sample preparation and analysis: aflatoxin and fumonisins in corn using a BenchMate Workstation

*Lynn Jordan, Zymark Corporation, Zymark Center, Hopkinton, MA 01748; Thomsen J. Hansen and Nancy A. Zabe, VIACAM L.P., Watertown, MA 02172*

Many major crops can be effected by toxic fungal metabolites known as mycotoxins. Two such mycotoxins are aflatoxin and fumonisin. Mycotoxins are of concern because they can impair human health and cause economic loss in livestock through disease and reduced production efficiency. The fungi that produce mycotoxins can invade the food and feed supply in the field, in processing, and in transport or storage. Several factors influence mycotoxin production by fungi, including substrate, moisture, temperature, pH, and stresses such as drought and associated growth of other microbes.

Fumonisin B1 is a mycotoxin produced by fusarium moniliforme, a frequent (almost universal) inhabitant of corn. Fumonisin analysis poses two problems: isolation of the mycotoxin from the sample matrix, and detection. Previously developed analytical methods for aflatoxins were based on immobilized antibodies. Antibodies for fumonisin B1 were produced and immobilized to make immunoaffinity columns for isolation of fumonisin B1 from corn. Derivitization through the primary amino group is necessary prior to HPLC analysis because fumonisin B1 contains no UV chromophores.

This paper illustrated the sample preparation and analysis for determination of aflatoxins and fumonisin B1 in corn. One sample extract can be prepared, and used for isolation of aflatoxins and fumonisin B1 by two similar methods. HPLC analysis is used for final determination of total level of aflatoxin or fumonisin.

### Automated cleanup and analysis of Lanolin for selectrd USP pesticides using a Zymark BenchMate Workstation

*Timothy Dame, Gordon Hamilton and Alison Bodkin, Energy and Environmental Engineering, Inc., Somerville, MA*

An automated method for the gel-permeation chromatographic separation of pesticides in lanolin utilizing a Zymark BenchMate robotic workstation was presented. Lanolin, a waxy substance from the wool of sheep, may contain trace levels of pesticides. Solvent dilution and subsequent gas chromatographic (GC) analysis do not achieve sufficiently low detection limits due to matrix interference from the lanolin. Gel-permeation chromatography (GPC) is commonly used for the separation of biomolecules and has been increasingly employed in the cleanup of environmental samples for analysis by gas chromatography (GC) and mass spectrometry (MS). GPC removes higher molecular weight interferences, thereby preventing unwanted accumulation of material in the GC injection port, lowering detection limits in complex matrices, and extending GC column life expectancy. Lanolin, a high molecular weight substance, lends itself well to automated sample processing using the BenchMate GPC Workstation.

Samples of lanolin were spiked with varying concentrations of selected USP chlorinated pesticides and subjected to sample processing and GPC cleanup using the Zymark BenchMate. A gravimetric audit trail was provided by the system to accurately track all liquid handling. After sample elution and concentration, the extracts were analyzed on a Hewlett Packard 5890 Series II dual-column gas chromatograph with electron-capture detection (ECD). Quality control results and chromatograms are presented that show the successful separation of pesticides from the lanolin matrix. Accurate quantification of the pesticides and high surrogate recoveries were obtained with the BenchMate system while minimizing technician labour and providing an electronic audit trail.

### The adaptation of a solid phase extraction method to the BenchMate and the Zymate XP

*Y. L. Tam and K. M. Hama, Syntex Research, Palo Alto, CA*

The analysis of mycophenolic acid and its glucuronide conjugate was transferred from a manual solid phase extraction (SPE) method to a Zymark XP robotic system equipped with two High Performance Solid Phase Extraction Pysections (HPSPE). The manual method used loosely packed 3-ml SPE columns with 100 mg of ODS packing. The sample was gravity-fed onto the column, followed by water rinse and finally eluted with methanol/acetate buffer, pH 4 (80/20, v/v). The manual method with slight modifications was initially automated to a Zymark BenchMate Workstation. Instead of gravity-feed onto the SPE column, the sample was loaded onto a commercially available 200 mg ODS SPE column at the rate of 0·02 ml/sec (1·2 ml/min). Sample was then rinsed with a 25 mM citric acid before eluted with the methanol/acetate buffer, pH4 (80/20, v/v) at the rate of 0·50 ml/sec (30 ml/min). The final extract was injected off-line for HPLC analysis. Modifications to the hardware and software controlling the HPSPE PySections were necessary for validation of the method. Sample throughput for the automated method is approximately 100 samples per 24 hours. Data generated from the automated method compared favourably with the data generated from the manual method.

### An automated method for the determination of mycophenolic acid and mycophenolic acid glucuronide in samples of human urine using a BenchMate Workstation

*M. L. Sung and K. M. Hama, Syntex Research, Palo Alto, CA*

Samples of human urine after thawing and centrifugation were processing automatically by the BenchMate Workstation for the determination of mycophenolic acid (MPA) and its glucuronide conjugate (MPAG). Urine samples were diluted serially and gravimetrically by the BenchMate with acetonitrile/water (10/90, v/v) by a factor of 10 for MPA and a factor for MPAG. An appropriate internal standard was thereafter added gravimetrically to an aliquot of each diluted urine sample and each treated sample was injected onto the HPLC-UV system with on-line data acquisition capabilities. The detection limits were 2.5 μg and 50 μg per ml of undiluted urine sample for MPA and MPAG, respectively. The precision (%CVs) of the BenchMate in performing the 1:10 dilution, the 1:50 dilution, and the addition of the internal standards exceeds 99%. For MPA the intra-assay CVs ($N = 4$) were $< 5·3\%$ and the inter-assay %CVs ($N = 4$) were $< 10·5\%$ except for the lowest standard curve point (0.25 μg/ml) for which the intra-assay %CV was 21·7%, For MPAG the corresponding intra-assay and inter-assay %CVs were $< 5·2\%$ and 11·4%, respectively. The accuracy of the method, represented by the ratio of found concentration to the nominal concentration (% recovery), ranged from 90·3% to 106·2% for MPA and 95·7% to 105·9% MPAG.

### Automated extraction of environmental compounds using solid phase extraction for GC/MS

*Margaret Marr, United Chemical Technologies, Horsham, PA*

Monitoring compounds of environmental concern has shown significant increase during the past few years. Pesticides, herbicides, polychlorinated biphenyls, polynucleated aromatic hydrocarbons, and 4,4′-methylenedianaline were extracted from drinking water on Worldwide Monitoring® Enviro-Clean extraction columns using the AutoTrace extraction system from Zymark Corporation. Concentrations as low as 10 μl were detected on the GC/MS. Sample/sorbent contact time is a crucial factor in obtaining optimum recoveries when using the AutoTrace.

Bonded phase extraction columns are less time consuming, solvent conservative and allow for high sample volumes. Sorbent masses are available from up to 10 000 mg in a variety of tube configurations. Each lot of sorbents is quality control tested to ensure that it will perform consistently.

The AutoTrace processes six samples at a time. This technology allows the user to set exact flow rates and uses controlled positive pressure to push fluid through solid phase extraction columns. Thus, results are more consistent and reproducible than with manual extraction methods.

### Robotic system for the preparation of inorganic samples for ICP, GFAA, and Hg analyses

*L. A. Kaplan, D. S. Layne, C. L. Horn, D. C. Yaworksy, Virginia Power System Laboratory, Chester, VA; and R. A. Stockton, SLR Systems, Richland, WA*

A general description and video tape of an automated hotplate digestion system capable of ICP, GFAA, and Hg inorganic sample preparation was presented, The Hewlett Packard ORCA based system can perform the EPA procedures shown below without operator intervention.

Variations from the exact EPA procedures are minor. Examples of the variations are:

(1) Use of a 300 ml beaker instead of a 250 ml Phillips beaker of BOD bottle.
(2) Determination of all volumes gravimetrically.
(3) Use of a 95 degree computer controlled hot block instead of a 95 degree water bath for the mercury digestions.

*Methods currently being performed*

| EPA Method # | Method title |
|---|---|
| 200.7 | Determination of Metals and Trace Elements in Water and Wastes by Inductively Coupled Plasma—Atomic Emission Spectrometry |
| 245.1 | Determination of Mercury in Water by Cold Vapor Atomic Absorption Spectrometry |
| 300.5 | Acid Digestion of Waters for Total Recoverable or Dissolved Metals for Analysis by FLAA or ICP |
| 3010 | Acid Digestion of Aqueous Samples and Extracts for Total Metals for Analysis by FLAA or ICP Spectrometry |
| 3020 | Acid Digestion of Aqueous Samples and Extracts for Total Metals for Analysis by GFAA Spectroscopy |
| 7470 | Mercury in Liquid Waste |

*Procedures requiring minor changes*

| EPA Method # | Method title |
|---|---|
| 200.2 | Sample Preparation Procedure for Spectrochemical Determination of Total Recoverable Elements |
| 200.3 | Sample Preparation Procedure for Spectrochemical Determination of Total Reoverable Elements in Biological Tissues |
| 200.8 | Determination of Trace Elements in Water and Wastes by Inductively Coupled Plasma-Mass Spectrometry |
| 245.5 | Determination of Mercury in Sediment by Cold Vapor Atomic Absorption Spectrometry |
| 7471 | Mercury in Solid of Semisolid Waste |
| 3050 | Acid Digestion of Sediments, Sludges and Soils |
| CLP SOW | Contract Laboratory Statement of Work IL2.0 |

Sample throughput varies dependent upon the procedure but ranges from 36 to 108 samples per day. All QC samples are prepared during the run as required by EPA procedures. Sample and reagent weights are recorded and available for operator inspection. Sample and digestate bottle identies are determined via bar codes.

### Quality assurance for a residue-screening robotic system

*Kenneth V. Miller, US Food and Drug Administration, New Orleans District Laboratory, New Orleans, LA*

The FDA District Laboratory in New Orleans is using a Zymark PyTechnology System to develop an automated method for screening fruits and vegetables for pesticide residues. The system includes capping, vortexing, solid addition, a liquid–liquid extraction, evaporation and GC-injection stations. As part of FDA's field laboratory Quality Assurance (QA) Program, a QA standard was written for the system. Elements of the standard include:

(1) A complete system description, including all modules and ancillary devices, table layout, MLS syringe dedications, PEC dedications and controller software.
(2) Standards for specific modules for applications software and for the execution of applications programs.
(3) A schedule for instrument maintenance and performance checks.
(4) Requirements for maintaining records of QA activities.

Standards for specific modules include balance accuracy, dispensing and aspirating accuracy and gas chromatograph performance (precision and linearity). Standards for applications software involve maintaining a list of unit operations for each applications program and periodically verifying execution of each of these in actual operation. Standards for the execution of applications programs include weight verification of critical operations, visual observation of input and output containers and critical examination of analytical instrumentation results.

The philosphy of the QA standard is to derive quality assurance from readily available system information and to require QA-specific testing only if such is not available to verify a specific aspect of the system's performance.

### Determination of total $^{14}C$ residue in soil, plant and animal tissue-automation of a biological sample oxidizer

*A. R. Vispetto and K. C. Kaya, ZENECA Ag Products, Metabolism Section, Richmond, CA; and R. N. Jones, ZENECA Agrochemicals, Environmental Science Department, Jealott's Hill Research Station, Bracknell, Berkshire, UK*

A Zymate II Laboratory Automated Combustion System was developed for the R. J. Harvey OX 300 and 500 Biological Oxidizers. Soil, plant and animal tissues are prepared and combusted. The $^{14}CO_2$ generated is used to calculate total $^{14}C$ residues in the tissues. Many samples are normally generated from radiolabelled metabolism studies, and the automated analysis eliminates the tedious, repetitive and manual operation of combustion.

Combustion is a common technique utilized for total residue analysis with radiotracers in pesticide metabolism studies. The Zymate II pours weighed and prepared samples from a 20 ml scintillation vial onto a quartz ladle for subsequent combustion in the Harvey system. The scintillation vial is held securely in a vial trapping station and filled with combustion cocktail for absorbing $^{14}CO_2$. The Harvey Oxidizer system was chosen for this automated application because of its relatively simple design. Automating the Harvey Oxidizer has alleviated the mundane operation of combustion and has released valuable manpower with the organization. This poster describes two automated biological material oxidizers operated by Zymark Laboratory robots.

### Quantitation of perfluorooctanoate in serum by gas chromatography/mass spectrometry using a Zymate II robot for automated sample preparation

*George E. Colaizy, James T. Wolter, James D. Johnson, 3M Company, Environmental Laboratory & Pollution Control, St. Paul, MN; and Pat Rethwill, Pace, Inc., Minneapolis, MN*

Perfluorooctanoate (PFO) is an anionic surfactant that is widely used in industrial formulations. As a manufacturer, there is interest in monitoring the blood levels of PFO in workers who may be exposed to this compound.

Quantitation of PFO can be accomplished by using tetrabutylammonium to ion-pair with free and bound perfluorooctanoate present in human serum. The ion-paired complex is extracted with ethyl acetate and then derivatized with benzyl bromide to form the benzyl ester. The benzyl ester can then be quantified using gas chromatography/mass spectrometry (GC/MS) in the selected ion mode.

The use of a robot to perform the extraction increases both the speed and accuracy of the extraction while virtually eliminating any change of laboratory workers being exposed to blood-borne pathogens. The phase aliquoting, extraction, and transport of the extract by a

Zymate II robot will be described. Quality control steps are included in the method. The GC/MS system and examples of standard curves were shown.

### Determination of racemic warfarin in human plasma by robotic sample preparation and high-performance liquid chromatography

*Dennis M. Garner, Henry J. Pieniaszek, Jr., S. Peter King, John E. Gray and Check Y. Quon, Drug Metabolism and Pharmacokinetics Section, The DuPont Merck Pharmaceutical Company, Stine-Haskell Research Center, Newark DE*

A sensitive, specific and rapid higher-performance liquid chromatographic method using a Zymark Zymate robotic system for sample preparation was developed for the determination of racemic warfarin in human plasma. Plasma samples were acidified and extracted with ethyl acetate. The organic extract was evaporated, reconstituted with mobile phase and chromatographed on a Zorbax C8 HPLC column with fluorescence detection. The quantifiable limits were 6.3 to 450 ng/ml using 1·0 ml of plasma based on precision and accuracy constraints of less than 15% coefficient of variation (%CV) and less than 15% difference, respectively. The intraday %CV ranged from 2·0% to 8·7%. The corresponding interday %CV ranged from 1·3%to 6·7%. The accuracy ranged from 1·4% to 6·7% difference. The recovery of warfarin from human plasma was 63 ± 8%.

The Zymate system performed reliable and consistent liquid/liquid extractions. It consisted of the following components: a Zymark XP robot with System V Controller, a general purpose hand, a refrigerated sample input storage rack, a Master Lab Station for dispensing and aliquoting, a custom tumble mixer, a centrifuge station, an evaporation station, a fully automated capping station, a vortex station, a weighing station, and an optical sensor for the detection of the liquid/liquid interface. The entire system was housed in a custom enclosure vented to the outside of the building which limited exposure to biohazards, organic liquids/vapours and mechanical hazards associated with the robotic arm. The system had the capacity to process 100 samples within an unattended analytical run. The chromatographic analysis was complete within 15 minutes.

A comparison of the manual versus robotic procedures was also shown. The validation results utilizing the robotic system compared favourably to the validation results of the manual method.

### Electronic sample and data management in a robotics laboratory

*S. Jennings, R. Von Culin and S. Conder, Bristol Myers Squibb, New Brunswick, NJ*

The Robotics Laboratory in Analytical R & D at New Brunswick has set up an electronic sample and data management system to increase productivity and data integrity for approximately 15 000 samples a year. Sample log-in is accomplished with a barcode reader and descriptive information on the samples is obtained from the stability database in electronic (ASCII) format. The sample information is distributed over a local area network to one of the eight PC controllers for the robotic assay system. Automated assays are started electronically by the system manager using the descriptive sample information. The assays are completed, results are calculated and a report is generated. Raw peak area data from all HPLC assays are transferred electronically to PC-based spreadsheet or custom program calculations for report generation. This eliminates tedious error prone transcription of data from one computer to the other. Reports suitable for use in the notebook are created along with an electronic facsimile of the report that can be used to transfer results to the stability database. This eliminates approximately one analyst-day per week slave to a terminal and reduces data management related errors.

### Implementation of graphical user interfaces and file structures for intelligent automation using Visual Basic

*Frank A. Settle, Jr, Daniel Y. Pharr, Christoffer Lagerholm, Department of Chemistry, Virginia Military Institute, Lexington, VA 24450; Lolo Lasida, Christopher Myers, Department of Mathematics and Computer Science, Virginia Military Institute, Lexington, VA 24450; Emily Knick and Robert Williams, Department of Chemistry, James Madison University, Harrisonburg, VA*

The design and implementation of a graphical user interface (GUI) and supporting file structure for intelligent, automated systems was presented. The demonstration system performs different colorimetric determinations including orthophosphate, iron(II), and aluminium. Variations in the procedures demonstrate the system's flexibility and reliability. A Zymark System V equipped with a Milton-Roy 601 spectrophotometer performs the analysis.

The object oriented programming environment of Visual Basic provides: (1) an efficient user interface to the automated system, (2) smooth transfer of information and data among system components, (3) pre- and post-run calculations, (4) transfer of data to and from spreadsheets using dynamic data exchange (DDE), (5) creation of files for transferring data to LIMS systems and (6) preparation of final reports. The GUI employs standard Windows formats whenever possible. The direction and file structures and designed for easy access to methods, calibration information, and results from unknowns.

Initially, the GUI combines information from the analyst with procedural variables from the method file, transfers the results to the EasyLab system control programs, and initiates a run. Windows displays monitor the procedure. Procedures include the determination and storage of calibration parameters as well as determination of standards after a predetermined number of samples have been run. This approach demonstrates the encapsulation, transfer, and performance of standard methods of automated analysis.

### PK-IMS, a pharmacokinetics information management system

*John Gray, Anna Davidson, Cynthia Robinson, Sharon Diamond, Vanessa Peterman, The DuPont Merck Pharmaceutical Company, Drug Metabolism and Pharmacokinetics Section, Stine-Haskell Research Center, Newark, DE; and Gregg Noll, GRQ Software Associates Inc., Exton, PA*

As laboratory robotics and automation increase the number of samples extracted for pharmacokinetic analysis, the rate limiting step in a lab can become the calculation and management of the pharmacokinetic data. PK-IMS is a menu driven data management system for pharmacokinetic analysis. The program is designed to save data analysis time, facilitate auditing, and reduce computing costs. The program is written in RPL and operates within the RS/1 (BBN Software Products Corp., Cambridge, MA) data management system on a VAX computer.

The system receives tables of chromatographic data as peak heights, peak areas or concentrations. The system can calculate drug concentration and determines a variety of pharmacokinetic parameters (i.e. t1/2, AUC, CL, V, etc.). Tables and graphs required for both preclinical and clinical pharmacokinetic studies are automatically generated. The user controls selection of data points for standard curve analysis and determination of half-life. Data changes are more easily made through the program's linked tables. Data integrity is assured through the use of computer validation procedures. The program structure also allows for further enhancements such as linking to other data or chromatographic systems and performing additional pharmacokinetic calculations.

### Alternative approach to control a Zymark robot

*Pierre B. Monnet, Thierry Dabin and Lionel Drugeault, Rhone-Poulenc Rorer, Vitry-Alfortville Research Center, Vitry-sur-Seine, France*

Since 1987 the external control of Zymate peripheral devices has been interesting many laboratory designers. Gary W. Kramer opened the way with the remote control of the Z830 PEC, Z510 MLS and Z300 INST. However, neither Zymark Corporation nor any publisher showed how to operate the direct control of a Zymate Robot by an external computer. Such an ability would widen the application area for this laboratory robot.

The authors described the hardware modifications to enable the use of a standard RS232 serial interface which operates an external clock lead. These modifications require a simple converter to adapt the interface signal levels.

Furthermore, the authors introduced the decoding and analysis of the commands and messages protocols used to control all the capabilities of the robot. The lack of information about the communication protocol between the controller and the robot led us to determine a device to capture and display the exchanged messages.

The Zymate systems which are able to fit these modifications, and the trial results to show their effects on the robot by increasing the robot's performance were given.

Finally, the authors examined the advantages of this way of controlling the laboratory robot, over the classic one. Some of the contributions from this device are the multi-robot control with multi-task software, the management of the overlap area in a multi-robot system and the opportunity to mix dissimilar devices in order to enhance systems which were previously bounded.

### Interfacing the Whatman UniPrep filter system to a laboratory robot

*David Allen, Bristol-Myers Squibb, Princeton, NJ*

The UniPrep filter system is a rapid and convenient substitute for manually operated syringe filters. With a slight modificiation to the One-Shot filter processor, the system can also be used to filter solutions in a robotic environment. This type of filtration provides an economical and fast alternative to centrifugation for the sequestering of particulates prior to chromatographic injection.

In an example application, the UniPrep system was used in a solid phase extraction of biological materials. Using the filter barrel to collect the extraction eluate permitted evaporation, reconstitution, and filtration to be performed in one vessel. Eliminating centrifugation reduced sample preparation time by 10 minutes. In addition, routine maintenance on a HPLC was significantly reduced, and the volume of bio-hazardous waste produced by the procedure decreased by 20%.

Specifics on modifications to the One-Shot processor and solid phase extraction station were given, along with details on using the filter barrel in Zymark's Py-section TurboVap, designs for a Pysection filtration module, and programming code.

### Automation of Tretinoin cream preparation and light-obscuring precautions

*Kelly Johnson, Bernice Medwick, Richard Young and Phillip Lane, Robert Wood Johnson Pharmaceutical Research Institute, Raritan, NJ*

This poster described a pyrobotic program developed to prepare Tretinoin cream. Lancer syringes were filled with the Tretinoin cream and weighed into 50 ml centrifuge tubes. Samples were then dissolved and diluted in the sample diluent. Special light-obscuring precatuions were made to ensure that samples are prepared in a controlled environment to complete yellow light with the appropriate security measures.

### Automated dialyzer testing system

*Alan E. Borgwardt, Linda M. Custer and David R. Barnes, W. R. Grace & Company, 7379 Route 32, Columbia, MD*

An automated test system has been developed at the research division of W. R. Grace & Company to improve the accuracy and speed with which kidney dialysis membrane cartridges can be tested for hydraulic permeability, ultrafiltration coefficient, and solute clearance. To date, three identical systems have been installed at various Grace research facilities.

The system is comprised of elements for flow measurement and control, pressure measurement, conductivity measurement, and flow path selection. Flow is measured by analogue outputs from Coriolus mass flow meters and by dynamic serial output from a digital balance. Flow is controlled by serial commands to servo-speed-controlled tubing pumps. Pressure is determined by analogue output from differential pressure transducers. Conductivity is monitored by conditioned analogue signals from platinum conductivity probes. Flow path selection is accomplished by solenoid actuated pinch valves activaated by optically isolated digital relays. All devices are supervised by a Macintosh personal computer equipped with expansion cards for data acquisition and control and custom software written in the LavView 2 (National Instruments, Austin, TX) environment.

This system replaces a tedious, manual testing procedure that involved attention of a full-time attendant and has improved the accuracy and precision of results. Attendant operation is required to prepare reagents and prime all fluid lines in preparation for testing; however, measurement and control for each test is then performed automatically by the system with no operator intervention. Quality of permeability and ultrafiltration coefficient data is assessed by correlation coefficient of the pressure-versus-ultrafiltrate-flow relation, while quality of clearance data is assessed by closure of mass balance between clearances measures on blood and dialysate sides of the membrane.

### Mobile laboratory workstations

*Albert Marcus Morrishow, Department of Pharmacology, Mount Sinai School of Medicine, New York, NY*

Workstations that the scientist, engineer, student, or technical person can carry in pockets, and utilize while stitting, standing, or walking, are evolving into pivotal control, data acquisition, and computation task tools. Currently the sub-notebook, palmtop, and handheld computers control and gather data at base and/or field laboratory instruments. Established and evolving standards for the mobile workstations are ensuring the data and peripherals will be interchangeable among both computing and non-computing instruments. The workstations will be networkable, present intuitive user interfaces, contain multiple numbers and types of communication ports, utilize upgradable industry standard processor architectures, and implement industry standard operating systems and application development languages.

### The development of an automatic fill-up station for a sample preparation robot

*Takashi Koyama, Shigeyuki Koike, Toshikazu Ogawa, Toyoki Sugiyama, Moriaki Higo, Lion Corporation, Analytical Research Center, Tokyo, Japan; Shigetoshi Sekiyama, SIC Corporation, Tokyo, Japan; and Toshiyuki Hobo, Faculty of Technology, Tokyo Metropolitan University, Tokyo, Japan*

In many quantitative analyses, it is necessary to accurately control the volume of the sample solution. When electric chemical or spectrophotometric methods are used, it is essential to fill the sample exactly to a specified volume with a volumetric flask. The authors have developed a workstation combined with a Zymate System. The workstation is used for the automatic filling-up operation; using a laser beam the workstation detects a scale line and the surface of the solvent in the 50 ml test tube.

### A robotic system for cleaning automated melt index cartridges

*Ronald D. Jones, Phillips Petroleum Company, Bartlesville, OK*

Phillips Petroleum Company has developed a set of workstations which enable a Zymark robot to clean cartridges used by automated melt index machines. Using these workstations (which occupy only a small portion of the total workcell), the robot extracts a cartridge's orifice/residual polymer/piston core, then cleans its interior surface using disposable gun patches.

### Thin film tensile testing robotic system

*P. D. Hazelwood, D. W. Smith, L. A. Gowrie, Dow Chemical Canada, Sarnia, Ontario, Canada; and K. Dame, Instron Corporation, Canton, MA*

An automated system for the tensile testing of thin film samples has been developed for use in the R & D Plastics Fab & Testing Lab. The system consists of a Zymark XP robotic arm and System V controller working in conjunction with an Instron Model 4206 tensile machine. Some of the features include a custom film storage and retrieval rack, and automated specimen disposal.

The various types of film that have been processed successfully include: high density film, 8 linear low density film (thick and thin), low density film standards, stretch and cling film and heat seal samples. The data collected on film standards showed the system to operate with reduced variability compared to the current manual method.

The system runs unattended and is capable of processing 30 specimens per hour.

### Automation of the iodine–amylose method for retained thiosulphate in film

*Maureen S. Kaltenbach and Lance Burlingame, Eastman Kodak Company, Chemicals Quality Services, Rochester, NY*

Thiosulphate is the major component of photographic processing fixer solutions. The thiosulphate that remains in processed film after the final wash step will adversely affect image stability. One method used to determine the amount of thiosulphate retained in processed film is the spectrophotometric iodine-amylose method.

The iodine–amylose method involves four time-controlled steps. First, thiosulphate is extracted from a film strip. Second, formaldehyde and buffer are added to the extract to react with any extracted sulphiate, which would interfere in the determination. Third, solutions of iodide, iodate, amylose, and buffer are combined to form a controlled amount of blue-coloured iodine–amylose complex. Finally, the extract mixture is added to the iodine–amylose complex, where the thiosulphate reacts with iodine in the complex, reducing the amount of blue-coloured complex. A blank solution is prepared by following all steps except for the first step, so the blank does not contain any thiosulphate. The absorbance of the sample reaction mixture is measured and subtracted from the absorbance of blank solution. The retained thiosulphate is calculated from the absorbance difference by using a calibration equation generated using thiosulphate standards analysed by the same method.

The method is labour-intensive and time-consuming, with each determination requiring about 25 minutes and involving careful timing for reagent additions. Consequently, the procedure has relatively poor precision. With slight modification, the method has been automated using a Zymark BenchMate workstation equipped with a UV/Visible interface. The automated procedure virtually eliminates analyst intervention, thus improving method precision and reducing laboratory costs. The presentation discussed the intstrumental configuration, method optimization and precision studies, and manual-to-automated method comparison results.

### The liquid–liquid interface: a conductivity based phase boundary detection system

*Norman E. Fraley, Jr., Express Analytic, Downers Grove, IL*

Sampling from the bottom of a liquid containment vessel is a routine operation for the laboratory robot. When the method calls for collecting the upper layer of a bi-phase system, the task becomes more difficult. In a method where the lower phase has a variable volume, finding the phase boundary is a formidable task. This presentation described a system for sampling the layers and moving the sampling needle to find the variable phase boundary to ensure accurate sample collection.

### Virtual racks

*William R. Kew, Express Analytic, Downers Grove, IL*

'Virtual racks' are a creative way of programming to increase the throughput of a robotic system by eliminating ramp times. The programming allows the robot to finish all samples in the rack, *including any added during the run*, and then stop automating.

First, the robot checks for containers in the primary rack. Second, an equation based on working sample is used to adjust the rack index (in each put and get program). Third, in the top level program, the volume is set to zero the first time a container is picked up. This is necessary to prevent false warnings of impending overflow on rack positions that are being reused.

### Automated data handling of mercaptan analysis in natural gas

*Neal Coticelli, Laboratory Services, Engineering and Technical Services Department, Brooklyn Union Gas Company, Brooklyn, NY*

Because natural gas is essentially odourless, the gas transmission and distribution companies inject the gas with compounds possessing a characteristic odour. Should a gas leak develop, this distinctive odour provides an early warning signal to gas customers.

Public utilities must protect the communities they serve, while preventing false reports of gas leaks. Using customized gas standards and special custom computer software, integrated to their chromatography data acquisition system, Brookly Union Gas has mechanized a procedure to determine the total concentration of odorant in natural gas samples.

By providing automated computations of odorant concentration, the custom software program produces accurate and timely quantitative reports without any tedious manual calculations. It also accepts information directly from the Gas Company's chromatography data acquisition system. This unique system has reduced paperwork and enhanced laboratory productivity.

